# iEKPD 2.0: an update with rich annotations for eukaryotic protein kinases, protein phosphatases and proteins containing phosphoprotein-binding domains

**DOI:** 10.1093/nar/gky1063

**Published:** 2018-10-31

**Authors:** Yaping Guo, Di Peng, Jiaqi Zhou, Shaofeng Lin, Chenwei Wang, Wanshan Ning, Haodong Xu, Wankun Deng, Yu Xue

**Affiliations:** Department of Bioinformatics & Systems Biology, Key Laboratory of Molecular Biophysics of Ministry of Education, College of Life Science and Technology, Huazhong University of Science and Technology, Wuhan 430074, China

## Abstract

Here, we described the updated database iEKPD 2.0 (http://iekpd.biocuckoo.org) for eukaryotic protein kinases (PKs), protein phosphatases (PPs) and proteins containing phosphoprotein-binding domains (PPBDs), which are key molecules responsible for phosphorylation-dependent signalling networks and participate in the regulation of almost all biological processes and pathways. In total, iEKPD 2.0 contained 197 348 phosphorylation regulators, including 109 912 PKs, 23 294 PPs and 68 748 PPBD-containing proteins in 164 eukaryotic species. In particular, we provided rich annotations for the regulators of eight model organisms, especially humans, by compiling and integrating the knowledge from 100 widely used public databases that cover 13 aspects, including cancer mutations, genetic variations, disease-associated information, mRNA expression, DNA & RNA elements, DNA methylation, molecular interactions, drug-target relations, protein 3D structures, post-translational modifications, protein expressions/proteomics, subcellular localizations and protein functional annotations. Compared with our previously developed EKPD 1.0 (∼0.5 GB), iEKPD 2.0 contains ∼99.8 GB of data with an ∼200-fold increase in data volume. We anticipate that iEKPD 2.0 represents a more useful resource for further study of phosphorylation regulators.

## INTRODUCTION

In eukaryotes, protein phosphorylation is one of the most studied post-translational modifications (PTMs); mainly occurs on specific serine, threonine or tyrosine residues of protein substrates; and participates in the regulation of almost all aspects of cellular activities, including cell proliferation, metabolism and cell death (1–4). Protein phosphorylation is a reversibly dynamic process that is regulated by protein kinases (PKs) that provide the modification as ‘writers’ and protein phosphatases (PPs) that remove substrate modifications as ‘erasers’ (2,4–6). In particular, numerous proteins containing phosphoprotein-binding domains (PPBDs) can recognize phosphoserine (pS), phosphothreonine (pT) or phosphotyrosine (pY) residues in specific short linear motifs (SLMs) as ‘readers’ (7–9). Such a writer-eraser-reader system ensures the fidelity of phospho-signalling *in vivo*. Dysregulation of the phosphorylation system is highly associated with human diseases, including neurodegeneration, immune deficiency and cancer (10–13). Thus, the identification and annotation of protein phospho-regulators is fundamental for better understanding of protein phosphorylation.

PKs are most studied protein phospho-regulators, and great efforts have been made for the identification and classification of PKs. In 1995, Hanks and Hunter first established a hierarchical classification system with four levels, including group, family, subfamily and individual PKs, according to conserved sequence and structural profiles of kinase catalytic domains (14). In 2002, Manning *et al.* further extended the classification scheme into a system containing 10 groups, 134 families and 201 subfamilies (4). They developed the best curated database Kinase.com (also called as KinBase), which first identified 518 human PK genes, and the latest release carefully curated and maintained PKs in 12 eukaryotes (4). Manning's system was widely adopted for the construction of a number of additional data resources, such as RTKdb (15), KinG (16), PKR (17), Kinomer (18), Kinannote (19) and our previously developed EKPD (20). Given that the classification of PKs at the subfamily level is highly difficult and labour-intensive, later studies mainly focused on the development of less curated but more comprehensive databases. For example, the Kinomer database identified and annotated putative PKs in 52 eukaryotes at the family level (18). In addition, a highly useful tool, Kinannote, was designed for the computational characterization of eukaryotic PKs (ePKs) in 36 species; however, atypical PKs (aPKs) were not considered (19).

Compared with PKs, relatively less effort has been made for the identification and classification of PPs and PPBD-containing proteins. In 2004, Alonso *et al.* first identified 107 putative human protein tyrosine phosphatases (PTPs) and classified them into four groups or classes mainly based on their catalytic domains (21). Later, a PTP database was developed to classify 601 PTP domains in 61 eukaryotes (22,23). In 2008, Kerk *et al.* considered both PTPs and protein serine/threonine phosphatases (PSPs) and computationally identified 150 and 148 PPs in *Arabidopsis thaliana* and *Homo sapiens*, respectively (24). In 2013, Li *et al.* constructed the human DEPhOsphorylation Database (DEPOD) (25), in which 223 human PPs and nonprotein phosphatases were integrated and structurally classified into 18 families based on the CATH (26) classification. Later, the updated DEPOD contained 239 human phosphatases as well as 387 protein and nonprotein substrates (27). Interestingly, it should be noted that a number of histidine phosphatase (HPs), which share a conserved catalytic core centered on a histidine residue, can function as PSPs or PTPs (2). For example, phosphoglycerate mutase 5 (PGAM5) acts as a PSP to dephosphorylate and activate MAP3K5/ASK1 (28), whereas testicular acid phosphatase (ACP4/ACPT) and prostatic acid phosphatase (ACPP) demodify pY residues in several members of the epidermal growth factor receptor (EGFR) family (2,5). Recently, from nine eukaryotes, Chen *et al.* manually curated and computationally identified 1425 PPs, which were classified into 10 protein folds (groups), 21 families and 178 subfamilies (6). For PPBDs, Gong *et al.* reported the PepCyber:P∼PEP database, which collected 337 known and potential PPBD-containing proteins and 1123 protein substrates a decade ago (29). A summary of publicly available resources on the collection, identification and classification of kinases, phosphatases and PPBDs was shown in Supplementary Table S1.

**Figure 1.**
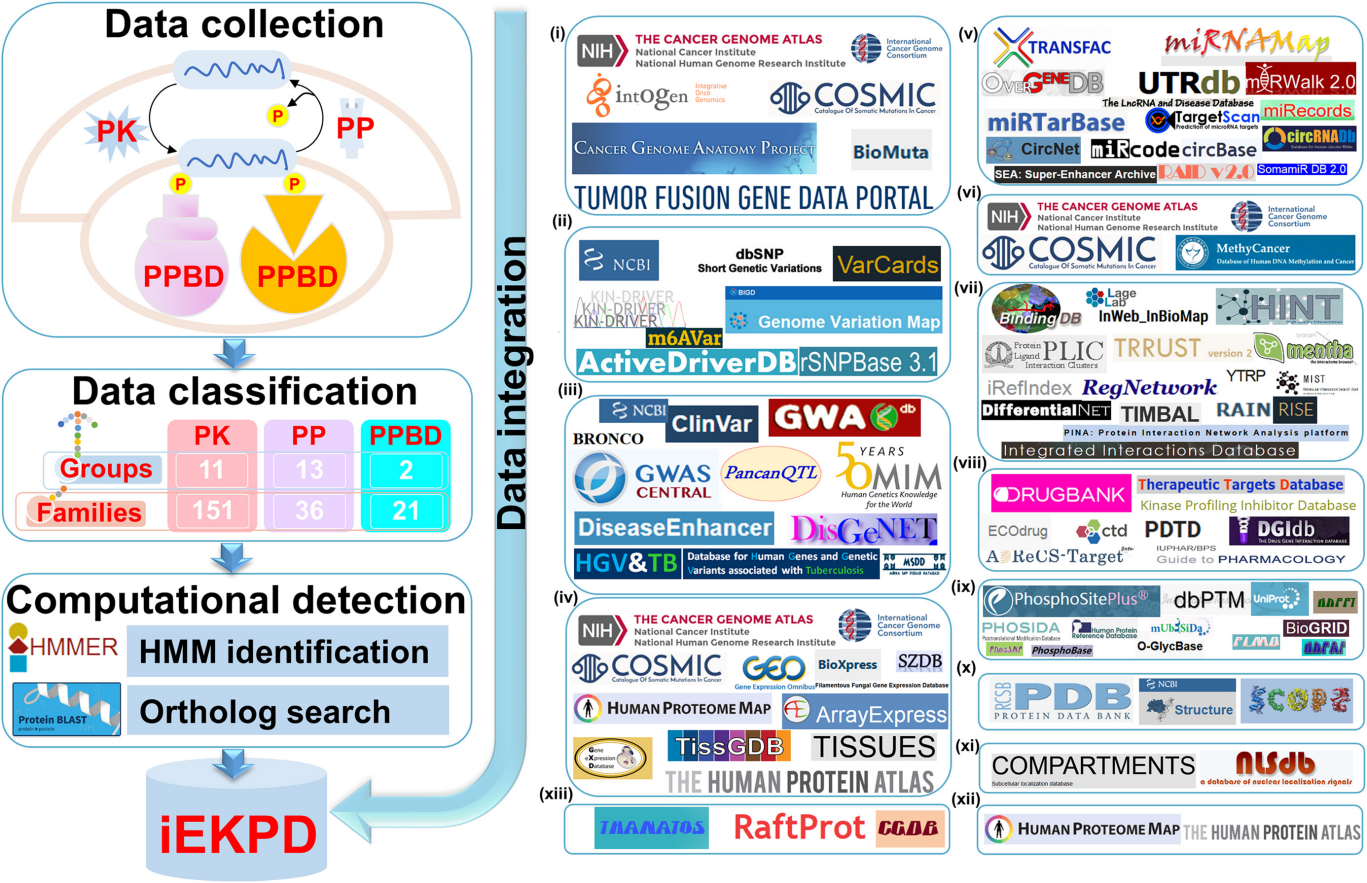
The procedure for the construction of iEKPD 2.0. First, we searched PubMed for experimental verified PKs, PPs and PPBD-containing proteins. We hierarchically classified all known PKs, PPs and PPBDs proteins to distinct groups and families and built HMM profiles for all available families. Then, we conducted HMM identification in 164 eukaryotes. For families without an HMM profile, we further performed orthologue detection using the reciprocal best-hit approach. In addition to basic annotation, we further integrated 100 public databases based on 13 aspects: (i) cancer mutations, (ii) genetic variations, (iii) disease-associated information, (iv) mRNA expression, (v) DNA and RNA elements, (vi) DNA methylation, (vii) molecular interactions, (viii) drug–target relations, (ix) protein 3D structures, (x) post-translational modifications, (xi) protein expressions/proteomics, (xii) subcellular localizations and (xiii) protein functional annotations.

In 2014, we reported a family-based database of EKPD 1.0, which contained 50 433 PKs and 11 296 PPs in 84 eukaryotes (20). At that time, PPBD-containing proteins were not included, and few annotations of phospho-regulators were provided. In this update, we first collected 1860 PKs, 439 PPs and 400 PPBD-containing proteins from the literature and hierarchically classified them into 26 groups and 208 families (Figure 1). Using the HMMER program (30), 129, 28 and 19 Hidden Markov Model (HMM) profiles were constructed for distinct PK, PP and PPBD families, respectively. Then, we conducted an HMM-based identification of potential phospho-regulators in 164 eukaryotes. For families without HMM profiles, we further performed an orthologous search of known regulators (Figure 1). In addition to gene/protein names, accession numbers, classification information, functional descriptions, protein/nucleotide sequences and other types of basic annotations, we further integrated knowledge from 100 publicly available databases that covered 13 aspects. In iEKPD 2.0, there were 197 348 known or potential phospho-regulators, including 109 912 PKs, 23 294 PPs and 68 748 PPBD-containing proteins in 164 eukaryotic species. Here, we confirmed that iEKPD 2.0 will be continuously updated to integrate newly discovered phospho-regulators and related information (Figure 1).

## CONSTRUCTION AND CONTENT

### Data collection

As previously described (20), we directly obtained 1855 classified and well curated PKs of five eukaryotes, including *H. sapiens, Mus musculus, Drosophila melanogaster, Caenorhabditis elegans* and *Saccharomyces cerevisiae*, from Kinase.com (4). Recently, it was demonstrated that a number of metabolic kinases, such as pyruvate kinase M2 (PKM2), phosphoglycerate kinase 1 (PGK1), ketohexokinase (KHK) isoform A (KHK-A) and hexokinase-1 (HK1) in *H. sapiens* as well as Hexokinase-2 (HXK2) in *S. cerevisiae*, can also functions as serine/threonine PKs (10). The five metabolic kinases were also included. Previously, we used a single keyword ‘phosphatase’ to search PubMed and manually curated 347 experimentally identified PSPs and PTPs from the literature. In this work, we chose the same approach and further collected 92 known PPs reported after 2014. For PPBD-containing proteins, we searched PubMed with multiple keyword combinations, such as ‘((phosphorylation) AND domain) AND bind’, ‘((recognize) AND phosphorylation) AND proteins’ and ‘(phosphorylation) AND protein interaction domain’. From the literature published after 2000, we curated 676 experimentally verified PPBDs in 400 proteins. The 123 known PPBDs curated in PepCyber:P∼PEP (29) were fully covered by our data set.

For the classification and genome-wide identification of phospho-regulators, we downloaded the complete proteome sequences of 164 eukaryotes, including 74 animals, 47 plants and 43 fungi, from Ensembl (release version 91, http://www.ensembl.org/), EnsemblPlants (release version 38, http://plants.ensembl.org/) and EnsemblFungi (release version 38, http://fungi.ensembl.org/), respectively (31). Because one gene can generate multiple variant protein sequences, we adopted the Ensembl Gene ID as the primary unique accession to avoid redundancy. For multiple isoform proteins of a gene, only the longest isoform was reserved for further analysis. Due to the low annotation quality of a considerable number of eukaryotic proteomes, we discarded all proteins containing at least one ‘X’ residue instead of a specific amino acid residue. For the proteome set of each organism, we used the CD-HIT program to clear redundant proteins with 100% identity (32). For known phospho-regulators, we retrieved their protein sequences from the non-redundant proteome libraries. The functional domains of the phospho-regulators were further extracted and verified from Kinase.com (4), Pfam (33), InterPro (34) and UniProt (35) to ensure data quality. From the results, we observed that one phospho-regulator can contain multiple different types of functional domains. For example, human PLK1 was simultaneously classified as a member of the Other/PLK family based on its N-terminal protein kinase domain (Pfam ID: PF00069), and a member of the (pS/pT)/PBD family based two Polo-box domains (PBDs, Pfam ID: PF00659) at its C-terminus (36). In total, we obtained 2643 non-redundant phospho-regulators, including 1860 PKs, 439 PPs and 400 PPBD-containing proteins (Supplementary Table S2).

### Genome-wide identification of PKs, PPs and PPBDs

**Figure 2.**
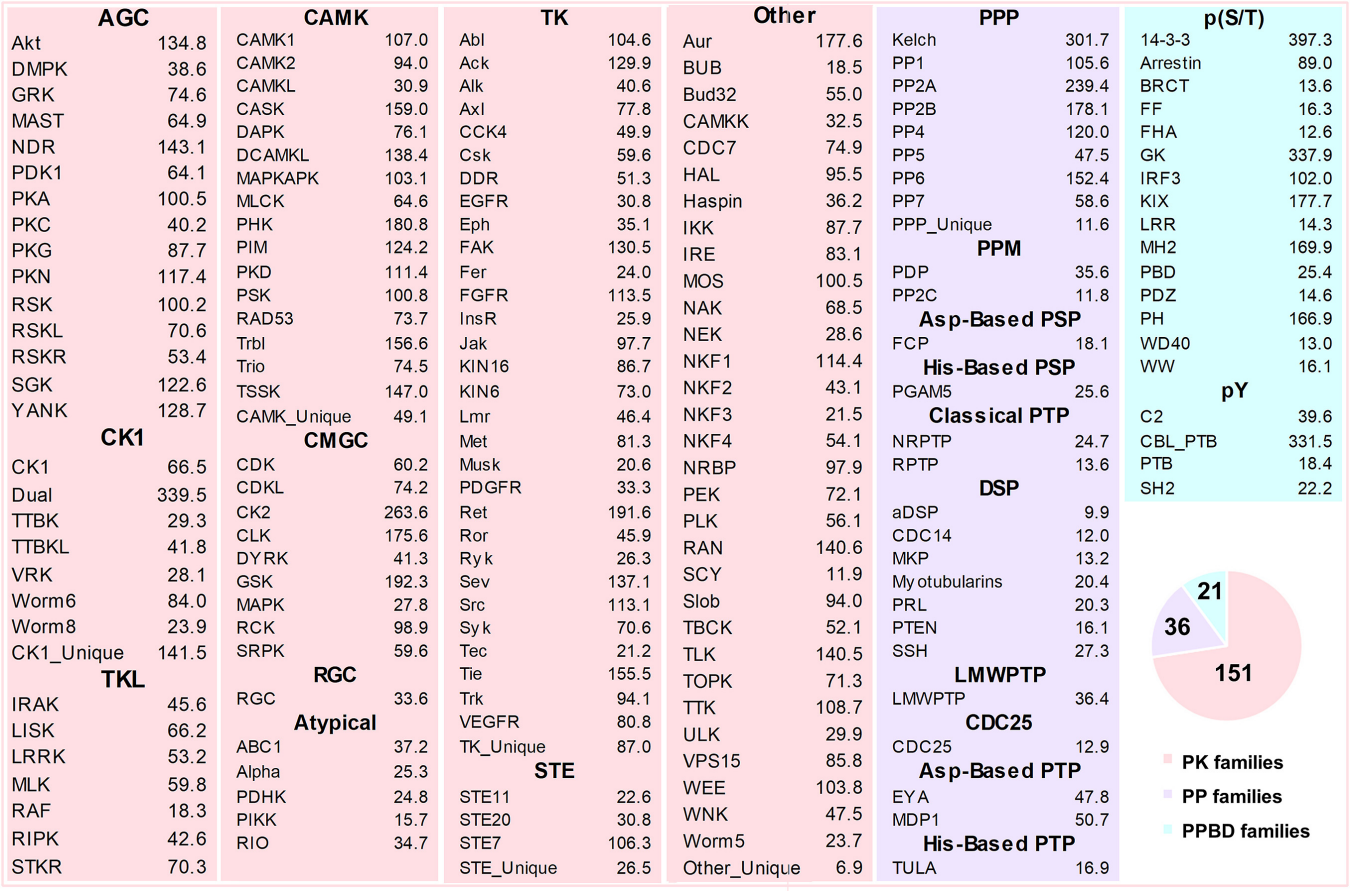
The classification of PKs, PPs and PPBDs together with cut-off values for all 176 HMM profiles. Log-odds likelihood scores are used as cutoffs for each family to avoid inconsistent results when the database is updated.

In EKPD 1.0, we classified curated PKs and PPs into 10 groups with 148 families and 10 groups with 33 families, respectively (20). In this work, PKs were classified into 151 families of 11 groups, whereas PPs were classified into 36 families of 13 groups (Figure 2). Also, PPBDs were classified into 21 families under the pS/pT group and the pY group (Figure 2). Details on the classification of known phospho-regulators was described in Supplementary Results.

As previously described (20), we retrieved protein sequences of functional domains for each PK, PP or PPBD family with ≥3 genes and separately multi-aligned them using MUSCLE (http://www.drive5.com/muscle/, version 3.8.31) (37). Then, the hmmbuild program in HMMER v3.1b2 package (http://hmmer.org/) was used to build 129, 28 and 19 HMM profiles for PK, PP and PPBD families, respectively (38). The default parameters were applied for MUSCLE and hmmbuild. Using the HMM profiles, the hmmsearch program of HMMER (38) was further adopted to search all protein sequences in 164 eukaryotic species for computational identification of potential PKs, PPs and PPBD-containing proteins. For each family, a log-odd likelihood score was selected as the cut-off value of hmmsearch (Figure 2). More details on the methodology of HMM-based predictions were present in Supplementary Results, whereas the performance was critically evaluated (Supplementary Results, Figure S1 and Table S3). All constructed HMM profiles can be downloaded from http://iekpd.biocuckoo.org/download.php.

For families without HMM profiles, we used the blastall program in the BLAST software package (39) and performed an orthologous search of known phospho-regulators with a classical approach of reciprocal best hits (RBH), which efficiently identifies orthologous pairs if two proteins in two different organisms are each other's best hit (40). Together with HMM- and orthology-based identifications, we identified 197 348 phospho-regulators, including 109 912 PKs, 23 249 PPs and 68 748 PPBD-containing proteins in 164 eukaryotic species. A heatmap of member genes in 11 PK groups, 36 PP families and 21 PPBD families across the 164 species were visualized using HemI (Supplementary Figure S2) (41), and detailed data counts were available for PKs (Supplementary Table S4), PPs (Supplementary Table S5), and PPBD-containing proteins (Supplementary Table S6).

### A multi-layer annotation of phosphorylation regulators

As a gene-centered database, iEKPD 2.0 provided the classification and domain profile information for each phospho-regulator as well as a variety of basic annotations obtained from Ensembl (31) and UniProt (35) databases, such as protein/gene names/aliases, functional descriptions, Ensembl/UniProt/GeneBank/RefSeq accession numbers, protein/nucleotide sequences, Kyoto Encyclopedia of Genes and Genomes (KEGG) and Gene Ontology (GO) terms, and domain/motifs. From UniProt (35), we also obtained the annotations of active sites for 10 569 unique phospho-regulators. In addition, the primary references of known phospho-regulators were obtained.

By integrating the knowledge of 100 additional databases, we further annotated 15 717 phospho-regulators in eight model organisms, including *H. sapiens, M. musculus, R. norvegicus, D. melanogaster, C. elegans, A. thaliana, S. pombe* and *S. cerevisiae* (Supplementary Table S7). These resources contained rich annotations that covered 13 aspects, including cancer mutations, genetic variations, disease-associated information, mRNA expression, DNA & RNA elements, molecular interactions, drug-target relations, protein 3D structures, PTMs, protein expression/proteomics, subcellular localizations and protein functional annotations (Supplementary Table S7). The data in each resource were carefully processed, and the details are presented in Supplementary Methods.

### USAGE

**Figure 3.**
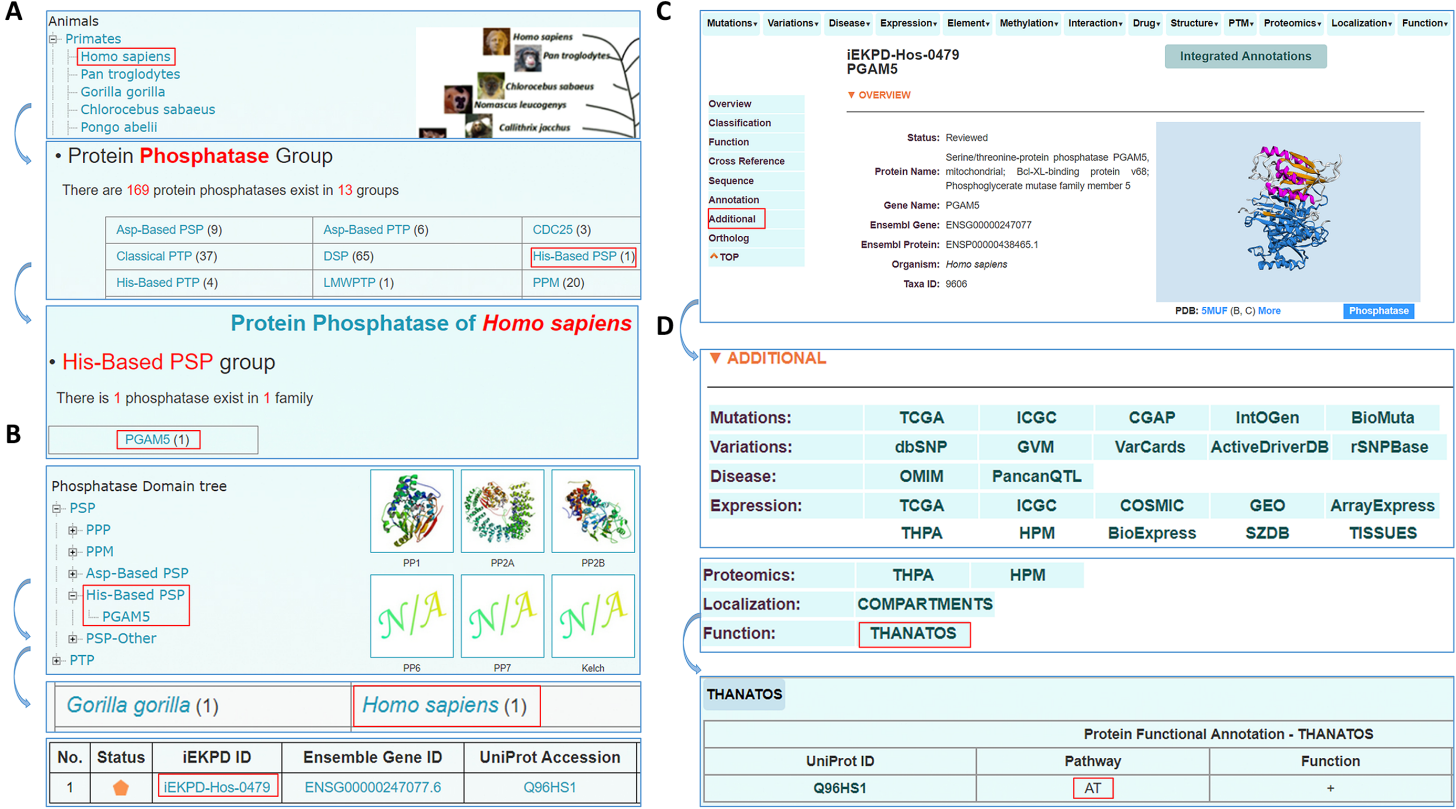
Usage of iEKPD 2.0. (**A**) Browse by species. (**B**) Browse by classification. (**C**) Basic annotation page of human PGAM5. (**D**) Additional annotation covering 13 aspects of human PGAM5.

The online service of iEKPD 2.0 database was developed in a user-friendly manner. Here, we chose human PGAM5, a member of the His-Based PSP/PGAM5 family, as an example to describe the usage of iEKPD 2.0. Two options, including ‘Browse by species’ and ‘Browse by classifications’, were implemented for browsing the data in iEKPD 2.0 (Figure 3). In the option of ‘Browse by species’, the Ensembl taxonomic categories were listed on the left side, whereas the phylogenetic relationships of the eukaryotic species in Ensembl were diagrammed in the right side (Figure 3A) (31). Users can click ‘*Homo sapiens’* to view all PK, PP and PPBD groups in *H. sapiens* (Figure 3A). By clicking ‘His-Based PSP’, the ‘PGAM5’ family under the group can be viewed. Then, users can click ‘PGAM5’, and a brief summary of human PGAM5, such as ‘Status’, ‘iEKPD ID’, ‘Ensemble Gene ID’, ‘UniProt Accession’ and ‘Gene Name’, are presented in a tabular format (Figure 3B). In iEKPD 2.0, all experimentally verified phospho-regulators were denoted as ‘Reviewed’ and marked with an orange pentagon, whereas computationally identified proteins were marked with a grey pentagon as ‘Unreviewed’ (Figure 3B). In addition, using the option of ‘Browse by classifications’, the user can click ‘PGAM5’ under the group of ‘His-Based PSP’ to browse all members of the His-Based PSP/PGAM5 family in eukaryotic species (Figure 3B). By selecting ‘*Homo sapiens*’ and ‘IEKP-Hos-0479’ (Figure 3B), the users can enter the basic annotation page of human PGAM5 (Figure 3C). In this page, users can find fundamental information, such as protein/gene names/aliases, family classification information, active site annotations, domain profiles, functional descriptions, cross references of accession numbers in public databases, protein/nucleotide sequences and other types of basic annotation (Figure 3C). To identify additional annotations, users can either click on the navigation bar at ‘Integrated Annotations’ or the label of ‘Additional’ (Figure 3C). The users can choose specific annotations, e.g. mRNA expression profiles in The Cancer Genome Atlas (TCGA) (42), and the results will be presented in a new window (Figure 3D). In addition, multiple search options, including ‘Simple Search’, ‘Batch Search’, ‘Advance Search’, ‘HMM Search’ and ‘BLAST Search’, were realized for querying the database.

## DISCUSSION

Protein phosphorylation is one of the most studied protein PTM and is involved in almost all aspects of cellular activities (2,4,20,43,44). In addition to PKs and PPs, PPBDs are also involved in protein phosphorylation signalling (43,44). Previously, we developed a hierarchical database of eukaryotic PKs and PPs, EKPD 1.0, containing 50 433 PKs and 11 296 PPs (Supplementary Table S8) (20). In this study, we further included PPBDs along with rich annotations retrieved from 100 public databases in iEKPD 2.0. In total, iEKPD 2.0 contains a dataset containing 197 348 protein phosphorylation regulators and is ∼99.8 GB in size, which is a >200-fold increase (Supplementary Table S8) compared with EKPD 1.0. A detailed comparison of EKPD 1.0 and iEKPD 2.0 is presented in Supplementary Table S8.

**Figure 4.**
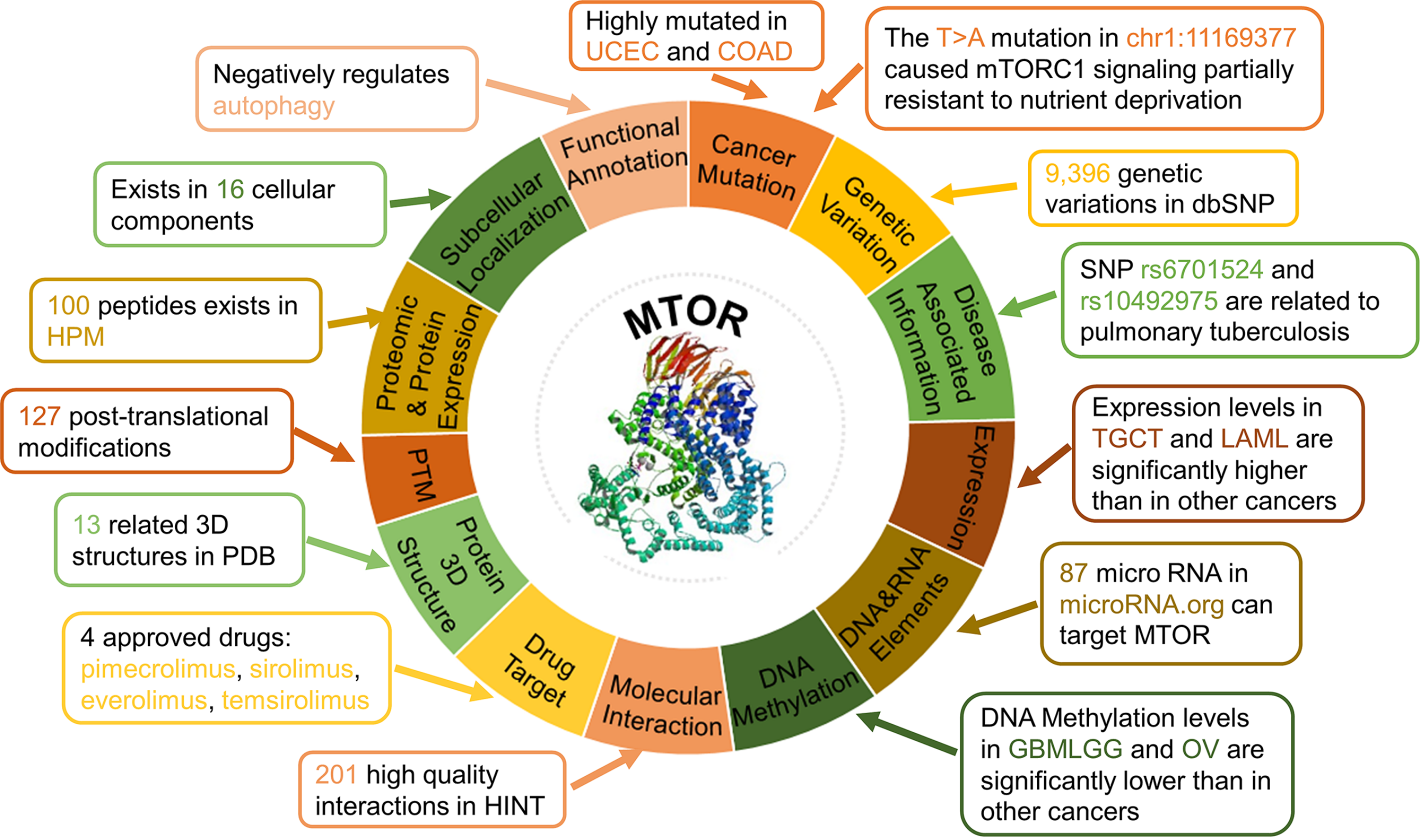
Overview of comprehensive annotations of human MTOR. Record numbers integrated from 78 additional databases are presented. A more detailed summary of 100 databases is provided in Supplementary Table S7.

Given the rapid progress in high-throughput technology, such as next-generation sequencing, the scientific community has produced a significant amount of biological data. Based on public available data, including manually curated and high-throughput data, numerous biological databases have been established. By integrating 13 aspects of additional annotations in 100 different databases, iEKPD 2.0 provides rich annotations for different aspects of genes. Such a comprehensive annotation provides useful information for researchers. For example, we integrated 420 cancer mutations from TCGA (42) for a human PIKK family kinase, MTOR (Figure 4), which is frequently mutated in a variety of cancers, especially uterine corpus endometrial carcinoma (UCEC) and colon adenocarcinoma (COAD) (Figure 4, Supplementary Figure S3A) (45). In particular, the T>A mutation in chr1:11169377, which resulted in a p.I2500F change on protein sequence, activates the PI3K–AKT–mTOR signalling pathway and causes mTOR complex 1 (mTORC1) signalling to be partially resistant to nutrient deprivation in cancer cells (Figure 4) (45). MTOR had 9396 genetic variations in dbSNP, and two SNPs, rs6701524 and rs10492975, are related to pulmonary tuberculosis (Figure 4) (46). In addition, two phosphorylation sites, S2448 and S2481, were closely related to multiple types of cancers (Supplementary Figure S3B). Expression levels in testicular germ cell tumours (TGCT) and acute myeloid leukaemia (LAML) are significantly increased compared with other cancers (Figure 4, Supplementary Figure S3C), whereas DNA methylation levels in glioma (GBMLGG) and ovarian serous cystadenocarcinoma (OV) are significantly reduced compared with other cancers (Figure 4, Supplementary Figure S3D). MTOR is targeted by 87 microRNAs in microRNA.org (Figure 4) and has 201 high-quality interaction partners in HINT. Moreover, four agents, including sirolimus (rapamycin), pimecrolimus (Elidel), everolimus and temsirolimus, has been approved by the FDA for the treatment of organ transplant rejection, and numerous clinical trials are in progress to assess sirolimus in cancer (Figure 4). MTOR has 13 related 3D structures in PDB and 127 post-translational modifications were identified (Figure 4). Furthermore, MTOR exists in 16 cellular components and negatively regulates autophagy (Figure 4) (47). For each integrated database, the iEKPD 2.0 database only presents the first 500 terms of information, and the full annotation can be download from http://iekpd.biocuckoo.org/download.php.

In summary, iEKPD 2.0 hosts 197 348 phosphorylation regulators, including 109 912 PKs, 23 294 PPs and 68 748 PPBDs in 164 eukaryotic species along with rich annotation of 13 aspects of genes, and the database contains ∼99.8 GB of data. We suggest that iEKPD 2.0 represents a useful resource for the community. Moreover, given the rapid progress in the field of protein phosphorylation, we will continuously update iEKPD 2.0.

## Supplementary Material

Supplementary Data
