# Supplementary material for: iEKPD 2.0: an update with rich annotations for eukaryotic protein kinases, protein phosphatases and proteins containing phosphoprotein-binding domains
Source: Nucleic Acids Res. 2018 Oct 31;47(Database issue):D344–50. doi: 10.1093/nar/gky1063 (PMC6324023; doi:10.1093/nar/gky1063)
Supplement: Supplementary Data [file gky1063_supplemental_files.zip › Supplementary Texts.docx]

Supplementary Data:

iEKPD 2.0: an update with rich annotations for eukaryotic protein
kinases, protein phosphatases and proteins containing phosphoprotein-binding domains

Yaping Guo^1,†^, Di Peng^1,†^, Jiaqi Zhou^1^, Shaofeng Lin^1^, Chenwei Wang^1^, Wanshan Ning^1^, Haodong Xu^1^, Wankun Deng^1,*^ and Yu Xue^1,*^

^1^Department of Bioinformatics & Systems Biology, Key Laboratory of Molecular Biophysics of Ministry of Education, College of Life Science and Technology, Huazhong University of Science and Technology, Wuhan 430074, China

***Running title*:** An updated database of protein phospho-regulators

^†^The authors wish it to be known that, in their opinion, the first two authors should be regarded as joint First Authors.

*To whom correspondence should be addressed.

Yu Xue, Tel: +86-27-87793903, Fax: +86-27-87793172, E-mail: [xueyu@hust.edu.cn](mailto:xueyu@hust.edu.cn).

Correspondence may also be addressed to Wankun Deng. E-mail: dengwankun@hust.edu.cn

**Supplementary** **Data** **Index**

**Supplementary Methods3**

**Supplementary Results30**

**Supplementary References34**

**Supplementary Figure S142**

**Supplementary Figure S243**

**Supplementary Figure S344**

**Supplementary Tables45**

**Supplementary Methods**

Besides basic annotation for each gene, iEKPD also integrates the knowledge of 100 additional databases for 15,717 phospho-regulators in eight model organisms, including *H. sapiens*, *M. musculus*, *R. norvegicus*, *D. melanogaster*, *C. elegans*, *A. thaliana*, *S. pombe* and *S. cerevisiae* (Supplementary Table S7). The data in each resource were carefully processed, and the details are presented below.

**1. Cancer Mutation**

*1.1) TCGA (*[*https://cancergenome.nih.gov/*](https://cancergenome.nih.gov/)*) (1)*

We downloaded all TCGA mutations provided by BROAD Institute (Oncotated calls, level 3, all available projects were downloaded, including adrenocortical carcinoma (ACC), bladder urothelial carcinoma (BLCA), breast invasive carcinoma (BRCA), cervical and endocervical cancers (CESC), cholangiocarcinoma (CHOL), colon adenocarcinoma (COAD), colorectal adenocarcinoma (COADREAD), lymphoid neoplasm diffuse large B-cell lymphoma (DLBC), oesophageal carcinoma (ESCA), glioblastoma multiforme (GBM), glioma (GBMLGG), head and neck squamous cell carcinoma (HNSC), kidney chromophobe (KICH), pan-kidney cohort (KICH+KIRC+KIRP) (KIPAN), kidney renal clear cell carcinoma (KIRC), kidney renal papillary cell carcinoma (KIRP), acute myeloid leukaemia (LAML), brain lower grade glioma (LGG), liver hepatocellular carcinoma (LIHC), lung adenocarcinoma (LUAD), lung squamous cell carcinoma (LUSC), mesothelioma (MESO), ovarian serous cystadenocarcinoma (OV), pancreatic adenocarcinoma (PAAD), pheochromocytoma and paraganglioma (PCPG), prostate adenocarcinoma (PRAD), rectum adenocarcinoma (READ), sarcoma (SARC), skin cutaneous melanoma (SKCM), stomach adenocarcinoma (STAD), stomach and esophageal carcinoma (STES), testicular germ cell tumours (TGCT), thyroid carcinoma (THCA), thymoma (THYM), uterine corpus endometrial carcinoma (UCEC), uterine carcinosarcoma (UCS),uveal melanoma (UVM). Dec., 2017) from BROAD Institude (http://gdac.broadinstitute.org/runs/stddata__ 2016_01_28/data/). Ensembl transcript IDs were used to map data from TCGA to iEKPD (Column entitled “Annotation_Transcript” in TCGA files). Finally, columns including "PRJ_code", "Chromosome", "Start_position", "End_position", "Strand", "Variant_Classification", "Variant_Type", "Reference_Allele", "Tumor_Seq_Allele1", "Tumor_Seq_Allele2", "Tumor_Sample_Barcode", "Matched_Norm_Sample_Barcode", "Genome_Change", "cDNA_Change", "Codon_Change" and "Protein_Change" were integrated.

*1.2) ICGC (*[*https://icgc.org/*](https://icgc.org/)*) (2)*

We downloaded all simple somatic mutations of ICGC release 26 from ICGC data portal (<https://dcc.icgc.org/releases/release_26/Projects>, Jan., 2018). Ensembl gene IDs (columns entitled “gene_affected” in ICGC data) were used to map mutations to iEKPD. Columns including "mutation", "project", "chromosome", "start", "end", "type", "ref", "alt", "consequence", "aa_mutation", and "cds_mutation" were extracted and integrated.

*1.3) COSMIC (*[*https://cancer.sanger.ac.uk/cosmic*](https://cancer.sanger.ac.uk/cosmic)*) (3)*

To ensure data quality, we downloaded censured cancer mutations from COSMIC in file “CosmicMutantExportCensus.tsv.gz” from COSMIC website ([https://cancer.sanger.ac.uk/cosmic /download](https://cancer.sanger.ac.uk/cosmic%20/download), v83). Ensembl transcript IDs were used as primary keys to map mutations to iEKPD. Columns including "Sample", "Mutation", "Mutation CDS", "Mutation AA", "Description", "Position" and "Strand" were integrated.

*1.4) CGAP (*[*https://cgap.nci.nih.gov/*](https://cgap.nci.nih.gov/)*) (4)*

The National Cancer Institute's Cancer Genome Anatomy Project (CGAP) presents an atlas of genes expressed during cancer development. We downloaded “Hs_GeneData.dat” and “Mm_GeneData.dat” from download page of CGAP (<https://cgap.nci.nih.gov/Info/CGAPDownload>, Jan., 2018). RefSeq nucleotide IDs were used to map data from CGAP to iEKPD. We integrated information including "UniGene", "Cytoband", "OMIM" and "SNP".

*1.5) IntOGen (*[*https://www.intogen.org/*](https://www.intogen.org/)*) (5)*

IntOGen catalogued multidimensional oncogenomic data for users. We downloaded mutation analysis file (“mutation_analysis.tsv”) for all available projects from IntOGen download page (<https://www.intogen.org/downloads>, Feb., 2018). Then, Ensembl transcript IDs were used as a primary key to map data from IntOGen to iEKPD, and columns including "Ref", "Alt", "Consequence", "Protein", "cDNA", "Chr", "Position" and "Cancer" were extracted.

*1.6) BioMuta (*[*https://hive.biochemistry.gwu.edu/biomuta*](https://hive.biochemistry.gwu.edu/biomuta)*) (6)*

We downloaded “BioMuta3.csv” from the BioMuta website (https://hive.biochemistry.gwu. edu/beta/biomuta/content/BioMuta3.csv, Jan., 2018). UniProt accessions were used as a primary key to map data from BioMuta to iEKPD, and columns including "BioMuta Index", "Position", "Ref", "Var", "Position(AA)", "Ref(AA)", "Var(AA)", "Cancer_type", "Function", and "PMID" were integrated.

*1.7) TumorFusions (*[*http://www.tumorfusions.org/*](http://www.tumorfusions.org/)*) (7)*

TumorFusions provided a data portal that catalogued cancer-associated transcript fusions. We downloaded “pancanfus.txt.gz” from its website (<http://www.tumorfusions.org/>, Jan., 2018). Gene symbols in "pancanfus.txt" were used to map data to iEKPD. We integrated information, including "Cancer", "Gene B", "Gene B Chr", "Gene B Strand", "Junction A", "Junction B", "Tier", "Frame" and "Centrality".

**2. Genetic varation**

*2.1) dbSNP (*[*https://www.ncbi.nlm.nih.gov/snp/*](https://www.ncbi.nlm.nih.gov/snp/)*) (8)*

First, all ASN1_flat files named with “ds_flat_ch1.flat” that provide a good deal of readable information about each SNP, and all rs_fasta files named with “rs_ch1.fas” that contain all available reference SNP (rs) sequence data presented in FASTA format were downloaded in the ftp site of dbSNP (November 11, 2016). All files are organized by chromosome number. In ASN1_flat file, rsID, Type alleles, het se(het), assembly, Gene Symbol, fxn-class, Residues, aa_position and chr-pos for each species were extracted. In rs_fasta file, the nucleic acid sequences that contain the SNP site with 15 upstream and 15 downstream bases were extracted. These SNPs were mapped to corresponding genes in Ensembl, and 12,732,688 non-redundant SNPs were finally obtained.

*2.2) GVM (*[*http://bigd.big.ac.cn/gvm/*](http://bigd.big.ac.cn/gvm/)*) (9)*

We downloaded all SNP profiles from ftp server of GVM for 14 species, including *Ailuropoda melanoleuca, Anas platyrhynchos, Bos taurus, Gallus gallus, Glycine max, Homo sapiens, Oryza sativa, Ovis aries, Populus trichocarpa, Solanum lycopersicum, Sorghum bicolor, Sus scrofa, Triticum aestivum,* and *Zea mays*. Because GVM only provided genomic positions of SNPs, here SnpEff (http://snpeff.sourceforge.net/), a tool for SNP annotation, was used to annotate GVM SNPs to genes to obtain Ensembl gene IDs. All missense variants were retained, and columns including "CHROM", "POS", "ID", "REF", "ALT", "Transcript", and "Peptide_alt" were integrated.

*2.3) VarCards (*[*http://varcards.biols.ac.cn/*](http://varcards.biols.ac.cn/)*) (10)*

VarCards catalogued the variant- and gene-level implications of given variants. We downloaded all SNP files from the download page of VarCards (Jan., 2018). Ensembl gene IDs were used to map data to iEKPD. Finally, we integrated information, including "Chr", "Start", "End", "Ref", "Alt", "Region", "Effect", "Mut Type" and "AA Change".

*2.4) ActiveDriverDB (*[*https://activedriverdb.org/*](https://activedriverdb.org/)*) (11)*

We downloaded “ad_muts.tsv” from the GitHub repository of ActiveDriver (<https://github.com/reimandlab/ActiveDriverDB.git>, Jan., 2018). Gene names were used to map data from ActiveDriverDB version 1.1 to iEKPD. Information, including "Gene", "Position", "WT_residue", "Mut_residue", "Cancer_type", and "Sample_ID", were retained in iEKPD.

*2.5) Kin-Driver (*[*http://kin-driver.leloir.org.ar/*](http://kin-driver.leloir.org.ar/)*) (12)*

The Kin-Driver database provided information for driver mutations in protein kinases. We downloaded “mutations_v82.txt” from download page (<http://kin-driver.leloir.org.ar/download.php>, Jan., 2018). UniProt IDs were used to map data to iEKPD. Finally, we integrated columns, including "Mutation", "Pfam Domain ID", "Rel Freq", "Abs freq", "Disease Freq", "Validation", "Mut type" and "Sample Count".

*2.6) m6AVar (*[*http://m6avar.renlab.org*](http://m6avar.renlab.org)*) (13)*

The m6AVar is a database of functional variants that are involved in m6A modification. We downloaded all available SNP profiles for 33 cancers, including ACC, BLCA, BRCA, CESC, CHOL, COAD, DLBC, ESCA, GBM, HNSC, KICH, KIRC, KIRP, LAML, LGG, LIHC, LUAD, LUSC, MESO, OV, PAAD, PCPG, PRAD, READ, SARC, SKCM, STAD, TGCT, THCA, THYM, UCEC, UCS and UVM, from the download page (<http://m6avar.renlab.org/download.html>, Jan, 2018,). Ensembl gene IDs were used as primary keys to map variants to iEKPD. Columns, including "m6A ID", "m6A Function", "Rs ID", "SNP Chr", "Start", "End", "Ref", "Alt", "Tumor", "m6A Change Pos", "m6A Sample" and "References", were retained for further integration.

*2.7) rSNPBase (*[*http://rsnp3.psych.ac.cn/*](http://rsnp3.psych.ac.cn/)*) (14)*

The rSNPBase 3.0 database provided SNP-related regulatory elements and SNP-based gene regulatory networks. We downloaded “rSNP_info_all.txt” from its website (<http://rsnp3.psych.ac.cn/downloadList.do>, Jan., 2018). We used Gene symbols to map data to iEKPD. Columns entitled "dbSNP ID", "Chrom", "Position", "Allele", "Region" and "Affected" were extracted and integrated.

**3. Disease-associated information**

*3.1) ClinVar (*[*https://www.ncbi.nlm.nih.gov/clinvar/*](https://www.ncbi.nlm.nih.gov/clinvar/)*) (15)*

First, we downloaded “clinvar_20170530.vcf” (~14.40 MB) file from ftp server of ClinVar (<ftp://ftp.ncbi.nlm.nih.gov/pub/clinvar/>, May, 2017). SnpEff (<http://snpeff.sourceforge.net/>) was used to annotate ClinVar records to the GRCh38.86 version of human genomics. Ensembl IDs were used as primary keys to map from ClinVar to iEKPD. Finally, columns entitled "RS_ID", "Chrom", "Pos", "Ref", "Alt", and "Phenotype IDs" were extracted for further integration.

*3.2) GWASdb (*[*http://jjwanglab.org/gwasdb*](http://jjwanglab.org/gwasdb)*) (16)*

We downloaded “gwasdb_20150819_snp_drug.gz” from GWASdb (Feb, 2018). Gene symbols were used to map data from GWASdb to iEKPD. Finally, columns entitled "CHR", "POS", "SNPID", "REF", "ALT", "P_VALUE", "P_VALUE_TEXT", "DURG_NAME", "DRUG_ANNO", "HPO_ID" and "PMID" were extracted for further integration.

*3.3) PTMD (*[*http://ptmd.biocuckoo.org/*](http://ptmd.biocuckoo.org/)*) (17)*

Recently, we developed PTMD for PTMs that are associated with human diseases. We downloaded “PTM-Disease association.zip” from PTMD (Feb, 2018). UniProt IDs were used to map data to iEKPD. Columns including "Disease", "PTM", "Type", "Residue", "Site" and "References" were integrated.

*3.4) OMIM (*[*http://omim.org/*](http://omim.org/)*) (18)*

We downloaded “mim2gene.txt” from the OMIM website (<https://omim.org/downloads/>, April, 2018). Ensembl gene IDs were used to map genes from OMIM to iEKPD. Information, including "MIM_number", "MIM entry type", "Entrez_ID", and "Approved gene symbol", were retained for further integration.

*3.5) MSDD (*[*http://www.bio-bigdata.com/msdd/*](http://www.bio-bigdata.com/msdd/)*) (19)*

The MiRNA SNP Disease Database (MSDD) documented experimentally supported associations among miRNAs, SNPs and human diseases. We downloaded "msdd.txt” from its website (<http://www.bio-bigdata.com/msdd/download.jsp>, Jan, 2018). We extracted information, including "Acc", "miRNA", "SNP", "Disease", "SNP position", "Allele", "Tissue", "Dysfunction Pattern", "Population", "Sample Size" and "References".

*3.6) DiseaseEnhancer (*[*http://biocc.hrbmu.edu.cn/DiseaseEnhancer/*](http://biocc.hrbmu.edu.cn/DiseaseEnhancer/)*) (20)*

DiseaseEnhancer provided a resource for human disease-associated enhancers. We downloaded “enhInfo-1.0.2.txt” from the download page of DiseaseEnhancer (<http://biocc.hrbmu.edu.cn/DiseaseEnhancer/JumpToDownload>, Jan, 2018). Gene symbols were used to map data to iEKPD. Columns entitled "DE ID", "Type", "Variation", "Chr", "Start", "End", "Allele", “Disease”, "Function" and "References" were extracted and integrated.

*3.7) BRONCO (*[*http://192.168.73.130/infos.korea.ac.kr/bronco/*](http://192.168.73.130/infos.korea.ac.kr/bronco/)*) (21)*

The Biomedical entity Relation ONcology COrpus (BRONCO) contained many variants and their relations with diseases. We downloaded "BRONCO_20151221.zip” from its website (Jan, 2018). Gene symbols were used to map data to iEKPD. We integrated information including "DE ID", "Type", "Variation", "Chr", "Start", "End", "Allele", "Function" and "References".

*3.8) HGVTB (*[*http://genome.igib.res.in/hgvtb/index.html*](http://genome.igib.res.in/hgvtb/index.html)*) (22)*

We downloaded "hgvtb.csv” from its website (Jan, 2018). Gene symbols were used to map data to iEKPD. We extracted information, including "rsid value", "var type", "pheno", "odds ratio", "reported pathogenecity", "concluded pathogenecity" and "References".

*3.9) DisGeNET (*[*http://www.disgenet.org*](http://www.disgenet.org)*) (23)*

DisGeNET provided human disease-associated genes and variants. Eleven TSV files, including "all_gene_disease_associations.tsv.gz”, "all_gene_disease_pmid_associations.tsv.gz”, "all_variant_disease_associations.tsv.gz”, "all_variant_disease_pmid_associations.tsv.gz”, "befree _gene_disease_associations.tsv.gz”, "befree_variant_disease_associations.tsv.gz”, "curated_ gene_disease_associations.tsv.gz”, "curated_variant_disease_associations.tsv.gz” and "pubannotator_variant_disease.tsv.gz”, were download from its website (http://www.disgenet.org /web/DisGeNET/menu/downloads, Jan., 2017). Gene symbols were used to map data to iEKPD. We integrated information, including "Disease ID", "Disease Name", "Score", "Nof References", "Nof SNPs" and "Source".

*3.10) PancanQTL (*[*http://bioinfo.life.hust.edu.cn/PancanQTL/*](http://bioinfo.life.hust.edu.cn/PancanQTL/)*) (24)*

PancanQTL was dedicated to comprehensively providing cis-eQTLs (SNPs affect local gene expression) and trans-eQTLs (SNPs affect distant gene expression) in 33 cancer types, including ACC, BLCA, BRCA, CESC, CHOL, COAD, DLBC, ESCA, GBM, HNSC, KICH, KIRC, KIRP, LAML, LGG, LIHC, LUAD, LUSC, MESO, OV, PAAD, PCPG, PRAD, READ, SARC, SKCM, STAD, TGCT, THCA, THYM, UCEC, UCS, and UVM. We downloaded all cis-eQTLs and trans-eQTLs profiles from the PancanQTL website (<http://bioinfo.life.hust.edu.cn/PancanQTL/download>, Jan., 2018). Gene symbols were used to map data to iEKPD. Columns entitled "Cancer", "Trans/Cis", "SNP", "Chr", "Position", "Alt" and "Position" were extracted and integrated.

**4. mRNA expression**

*4.1) TCGA (*[*https://cancergenome.nih.gov/*](https://cancergenome.nih.gov/)*) (1)*

We downloaded all available mRNA expression profiles for 37 cancers, including ACC, BLCA, BRCA, CESC, CHOL, COAD, COADREAD, DLBC, ESCA, GBM, GBMLGG, HNSC, KICH, KIPAN, KIRC, KIRP, LAML, LGG, LIHC, LUAD, LUSC, MESO, OV, PAAD, PCPG, PRAD, READ, SARC, SKCM, STAD, STES, TGCT, THCA, THYM, UCEC, UCS, and UVM provided by BROAD Institute. Entrez IDs were used to map data from TCGA. “Project”, “Sample”, and “Expression” were retained for integration.

*4.2) ICGC (*[*https://icgc.org/*](https://icgc.org/)*) (2)*

We downloaded all available expression profiles for 31 ICGC projects, including BLCA-US, BRCA-KR, BRCA-US, CESC-US, CLLE-ES, COAD-US, GBM-US, HNSC-US, KIRC-US, KIRP-US, LAML-US, LGG-US, LIHC-US, LIRI-JP, LUAD-US, LUSC-US, MALY-DE, OV-AU, OV-US, PAAD-US, PACA-AU, PACA-CA, PAEN-AU, PRAD-FR, PRAD-US, READ-US, RECA-EU, SKCM-US, STAD-US, THCA-US, and UCEC-US, from the ICGC data portal (https://dcc.icgc.org /releases/release_26/Projects, Jan., 2018). Ensembl gene IDs (columns entitled “gene_affected” in ICGC data) were used to map mutations to iEKPD. Columns, including "project_code", "normalized_read_count", "raw_read_count", "icgc_sample_id" and "submitted_sample_id", were extracted and integrated.

*4.3) COSMIC (3)*

We downloaded “CosmicCompleteGeneExpression.tsv.gz” from the download page of COSMIC (https://cancer.sanger.ac.uk/cosmic/download, Oct, 2018). Gene symbols were used to map data from COSMIC. Columns entitled "SAMPLE_ID", "SAMPLE_NAME", "REGULATION", "Z_SCORE", and "ID_STUDY" were extracted and integrated.

*4.4) GEO (*[*http://www.ncbi.nlm.nih.gov/geo/*](http://www.ncbi.nlm.nih.gov/geo/)*) (25)*

The Gene Expression Omnibus is an international public repository for functional genomics data that allows users to download gene expression profiles. We downloaded 4,348 files from the FTP server of GEO (<ftp://ftp.ncbi.nlm.nih.gov/geo/>, Jan, 2018). Entrez IDs were used to map data to iEKPD. Columns entitled "ID Ref", "ID", "GSM IDs", "Values" and "Gene ID" were integrated.

*4.5) ArrayExpress (*[*http://www.ebi.ac.uk/arrayexpress/*](http://www.ebi.ac.uk/arrayexpress/)*) (26)*

ArrayExpress provided microarray-based gene expression data. We downloaded "allgenes_updown_in_disease_2.0.19.tab” from ArrayExpress website (Jan, 2018). Ensembl gene IDs were used to map data to iEKPD. We extracted information, including "Gene Name", "Experimental Factor", "Factor Value", "Experiment Accession", "Array Design Accession", "Expression" and "p Value".

*4.6) The Human Protein Atlas (*[*https://www.proteinatlas.org/*](https://www.proteinatlas.org/)*) (27)*

The Human Protein Atlas comprised a map of protein expression patterns in normal cells, tissues and cancer. We downloaded all available data files, including "normal_tissue.tsv.zip”, "pathology.tsv.zip”, "proteinatlas.trig.gz”, "proteinatlas.tsv.zip”, "proteinatlas.xml.gz”, "rna_celline.tsv.zip”, "rna_tissue.tsv.zip”, "subcellular_location.tsv.zip”, "transcript_rna_ celline.tsv.zip” and "transcript_rna_tissue.tsv.zip”, from its website (https://www.proteinatlas.org /about/download, Feb., 2018). Subsequently, Ensembl gene IDs were used to map data to iEKPD. Finally, columns, including "Ovary", "Urinary Bladder", "Skeletal Muscle", "Lymph Node", "Adrenal Gland", "Cerebral Cortex", "Seminal Vesicle", "Spleen", "Small Intestine", "Duodenum", "Placenta", "Skin 1", "Stomach 1", "Gallbladder", "Endometrium 1", "Rectum", "Kidney", "Lung", "Fallopian Tube", "Testis", "Colon", "Epididymis", "Breast", "Esophagus", "Bone Marrow", "Salivary Gland", "Parathyroid Gland", "Adipose Tissue", "Cervix", "Uterine", "Smooth Muscle", "Pancreas", "Tonsil", "Prostate", "Thyroid Gland", "Liver" and "Heart Muscle", were extracted and integrated.

*4.7) Human Proteome Map (*[*http://www.humanproteomemap.org*](http://www.humanproteomemap.org)*) (28)*

The Human Proteome Map (HPM) portal presented an interactive web resource for human proteome. We downloaded four files such as "HPM_gene_level_epxression_matrix_ Kim_et_al_052914.xlsx", "HPM_peptide_genes_Kim_et_al_052914.xlsx", "HPM_peptide_level_ expression_matrix_Kim_et_al_052914.xlsx" and "HPM_protein_level_expression_matrix_ Kim_et_al_052914.xlsx" from HPM website (<http://www.humanproteomemap.org/download.php>, Jan., 2018). Gene symbols were used to map data to iEKPD. We integrated information including "Fetal Heart", "Fetal Liver", "Fetal Gut", "Fetal Ovary", "Fetal Testis", "Fetal Brain", "Adult Frontal Cortex", "Adult Spinal Cord", "Adult Retina", "Adult Heart", "Adult Liver", "Adult Ovary", "Adult Testis", "Adult Lung", "Adult Adrenal", "Adult Gallbladder", "Adult Pancreas", "Adult Kidney", "Adult Esophagus", "Adult Colon", "Adult Rectum", "Adult Urinary Bladder", "Adult Prostate", "Placenta", "B Cells", "CD4 Cells", "CD8 Cells", "NK Cells", "Monocytes" and "Platelets".

*4.8) GXD (*[*http://www.informatics.jax.org/expression.shtml*](http://www.informatics.jax.org/expression.shtml)*) (29)*

The Gene Expression Database (GXD) collected mouse developmental expression information. We downloaded both "MRK_GXDAssay.rpt.txt” and “MRK_ENSEMBL.rpt.txt” from its website (<http://www.informatics.jax.org/mgihome/GXD/aboutGXD.shtml>, Jan., 2014). Ensembl gene IDs were used to map data from GXD to iEKPD. We extracted information including "Marker ID", "Marker Symbol", "Marker Name" and "MGI Assay Accession ID".

*4.9) BioExpress (*[*https://hive.biochemistry.gwu.edu/bioxpress*](https://hive.biochemistry.gwu.edu/bioxpress)*) (6)*

BioXpress provided gene/miRNA expression of disease samples. We downloaded “BioXpress_gene_differential_expression_v2.0.csv” from its website (Jan., 2018). UniProt accession number were used as the primary key to map data from BioXpress to iEKPD. We retained "log2 Fold Change", "p Value", "Significant", "Trend", "TCGA Cancer", "Cancer Ontology", "Patients" and "UBERON ID" for further integration.

*4.10) TissGDB (*[*https://bioinfo.uth.edu/TissGDB/*](https://bioinfo.uth.edu/TissGDB/)*) (30)*

TissGDB (Tissue specific Gene DataBase in cancer) provided a powerful retrieval resource for tissue-specific gene expression in cancer. We downloaded “Tissg_DEGs.txt” from the website of TissGDB (<https://bioinfo.uth.edu/TissGDB/download.html>, Jan., 2018). Subsequently, Gene symbols were used to map data from TissGDB to iEKPD. Finally, columns including "Cancer type", "Normal", "log2 (FC)", "p Value" and "FDR" were extracted and integrated.

*4.11) FFGED (*[*http://bioinfo.townsend.yale.edu/*](http://bioinfo.townsend.yale.edu/)*) (31)*

The filamentous fungal gene expression database (FFGED) provided comprehensive information for filamentous fungal gene expression. We downloaded all XML profiles (13420 files) from FTP server of FFGED. Ensembl gene IDs were used to map data to iEKPD. Columns entitled "Experiment ID", "Experiment Name", "Expression Variable" and "Expression Value" were integrated.

*4.12) SZDB (*[*http://www.szdb.org/index.html*](http://www.szdb.org/index.html)*) (32)*

SZDB provided a resource for gene expression data about Schizophrenia (SZ). We downloaded 9 files including "clusters.txt", "entries.txt", "gene.txt", "MD_CBC.txt", "PFC_MSC.txt", "Score.csv", "stages.txt", "STR_AMY.txt" and "V1C_STC.txt" from download page of SZDB (<http://www.szdb.org/download.html>, Feb., 2018). Gene symbols were used to map data to iEKPD. We retained columns including "Gene1 ID", "Gene1", "Pearson", "Stage" and "Cluster" for further integration.

*4.13) TISSUES (*[*https://tissues.jensenlab.org/*](https://tissues.jensenlab.org/)*) (33)*

We downloaded all expression profiles including "human_tissue_experiments_full.tsv", "human_tissue_knowledge_full.tsv", "human_tissue_textmining_full.tsv", "mouse_tissue_ experiments_full.tsv", "mouse_tissue_knowledge_full.tsv", "mouse_tissue_textmining_full.tsv", "pig_tissue_experiments_full.tsv", "pig_tissue_knowledge_full.tsv", "pig_tissue_textmining_ full.tsv", "rat_tissue_experiments_full.tsv", "rat_tissue_knowledge_full.tsv" and "rat_tissue_ textmining_full.tsv" from its website (<https://tissues.jensenlab.org/Downloads>, Jan., 2018). Ensembl protein IDs were used to map data from TISSUES to iEKPD. We extracted and integrated information including "Tissue ID", "Tissue", "Source", "Evidence/Z-score" and "Confidence".

**5. DNA & RNA Element**

*5.1) UTRdb (*[*http://utrdb.ba.itb.cnr.it/*](http://utrdb.ba.itb.cnr.it/)*) (34)*

UTRdb provided a powerful retrieval resource for 5' and 3' untranslated sequences of eukaryotic mRNAs. It also allowed users to search experimentally validated miRNA targets. We downloaded “UTRdb.7z” from download page of UTRdb (<http://utrdb.ba.itb.cnr.it/home/download>, Jan, 2018). Entrez gene IDs were used to map data to iEKPD. Columns entitled "UTR type", "ASPicDB", "UTRaspic", "RefSeq" and "Genome" were integrated.

*5.2) CircBase (*[*http://circbase.org/*](http://circbase.org/)*) (35)*

We downloaded “hsa_hg19_circRNA.7z” from its website (<http://circbase.org/cgi-bin/downloads.cgi>, Nov, 2017). RefSeq nucleotide IDs were used as primary key to map data to iEKPD. We integrated information including "circRNA ID", "Chr", "Start", "End" and "Strand".

*5.3) circRNADb (*[*http://202.195.183.4:8000/circrnadb/circRNADb.php*](http://202.195.183.4:8000/circrnadb/circRNADb.php)*) (36)*

We downloaded "circRNADb.zip” from circRNADb website ([http://202.195.183.4:8000 /circrnadb/resources.php](http://202.195.183.4:8000%20/circrnadb/resources.php), Jan., 2018). RefSeq nucleotide IDs were used to map data to iEKPD. We extracted information including "circRNA ID", "Chr", "Start", "End", "Strand" and "Samples".

*5.4) CircNet (*[*http://circnet.mbc.nctu.edu.tw/*](http://circnet.mbc.nctu.edu.tw/)*) (37)*

CircNet is a comprehensive repository of circular RNAs. We downloaded “CircNetData.zip” from CircNet website. RefSeq nucleotide IDs were used to map data to iEKPD. Columns including "Circ ID", "GI", "Position", "Express", "Entrez", "Category", "Strand" and "Refered Name" were extracted and integrated.

*5.5) Circ2Traits (*[*http://gyanxet-beta.com/circdb/*](http://gyanxet-beta.com/circdb/)*) (38)*

We downloaded "circRNA_info_all.txt” from Circ2Traits website (Jan., 2018). RefSeq mRNA IDs were used to map data to iEKPD. RefSeq nucleotide IDs were used to map data to iEKPD. We extracted information including "Chr", "Start", "End", "Name", "Score", "Strand", "Samples", "Unique Reads" and "Repeats".

*5.6) miRTarBase (*[*http://mirtarbase.mbc.nctu.edu.tw/php/index.php*](http://mirtarbase.mbc.nctu.edu.tw/php/index.php)*) (39)*

The miRTarBase provided an open retrieval resource for experimentally validated miRNA-target interactions. “mirtarbase.txt” was downloaded from miRTarBase (<http://mirtarbase.mbc.nctu.edu.tw/php/download.php>, Jan., 2016). We used “Target Gene (Entrez ID)” to map data from miRTarBase to iEKPD. Finally, Columns including "mirTarBase ID", "miRNA", "Species (miRNA)", "Target Gene", "Species (Target Gene)", "Experiments", "Support Type" and "References" were extracted and integrated.

*5.7) microRNA.org (*[*http://www.microrna.org*](http://www.microrna.org)*) (40)*

We downloaded miRNAs for *C. elegans, D. melanogaster. H. sapiens, M. musculus, R. norvegicus* from its website. Entrez gene IDs were used to map data to iEKPD. We extracted information including "Mirbase Acc", "miRNA Name", "Gene ID", "miRNA Alignment", "Alignment", "Gene Alignment", "miRNA Start", "miRNA End", "Gene Start", "Gene End", "Genome Coordinates", "Conservation", "Align Score", "Seed Cat", "Energy" and "mirSVR Score".

*5.8) TRANSFAC (*[*http://gene-regulation.com/pub/databases.html*](http://gene-regulation.com/pub/databases.html)*) (41)*

The TRANSFAC database provided transcriptional gene regulation information. We downloaded “gene.dat” from its website. Subsequently, Entrez IDs were used to map data from TRANSFAC to iEKPD. Finally, columns including "AC", "AS", "OS", "Entrez", "References" and "BS" were extracted and integrated.

*5.9) miRWalk (*[*http://zmf.umm.uni-heidelberg.de/apps/zmf/mirwalk2/*](http://zmf.umm.uni-heidelberg.de/apps/zmf/mirwalk2/)*) (42)*

The miRWalk is a comprehensive repository of microRNA-target interactions. We downloaded “miRWalk.7z” from miRWalk website. Entrez gene IDs were used to map data to iEKPD. Columns including "miRNA", "Gene ID" and "Pathway" were integrated.

*5.10) TargetScan (*[*http://www.targetscan.org/vert_71/*](http://www.targetscan.org/vert_71/)*) (43)*

TargetScan provided a valuable resource for placing miRNAs into gene-regulatory networks. We downloaded 4 files including "Conserved_Family_Info.txt", "Conserved_Family_Info_1.txt", "Conserved_Family_Info_2.txt", "Conserved_Family_Info_3.txt" and "Conserved_Family_Info_ 4.txt" from TargetScan website. Gene symbols were used to map data to iEKPD. We extracted information including "miR Family", "UTR ID", "UTR Start", "UTR End", "MSA Start", "MSA End" and "Seed Match".

*5.11) miRecords (*[*http://c1.accurascience.com/miRecords/*](http://c1.accurascience.com/miRecords/)*) (44)*

We downloaded "miRecords_version4.xls” from its website (http://c1.accurascience.com /miRecords/download.php, Jan., 2018). RefSeq mRNA IDs were used to map data to iEKPD. We extracted information including "miRNA Mature ID", "miRNA Regulation", "Reporter Target Gene/Region", "Target Site Position" and "References".

*5.12) miRNAMap (*[*http://mirnamap.mbc.nctu.edu.tw/*](http://mirnamap.mbc.nctu.edu.tw/)*) (45)*

We downloaded all miRNA-target relationship profiles including "miRNA_targets_aga.txt", "miRNA_targets_cel.txt", "miRNA_targets_cfa.txt", "miRNA_targets_dme.txt", "miRNA_targets_ dre.txt", "miRNA_targets_fru.txt", "miRNA_targets_gga.txt", "miRNA_targets_hsa.txt", "miRNA_targets_mdo.txt", "miRNA_targets_mmu.txt", "miRNA_targets_rno.txt" and "miRNA_targets_xtr.txt" from miRNAMap website (ftp://mirnamap.mbc.nctu.edu.tw/miRNAMap2, Jan., 2018). Ensembl transcript IDs. were adopted as primary keys to map data to iEKPD. We retained information including "Mature miRNA", "Ensembl Transcript ID", "Target Start", "Target End", "miRNA 3-5", "Alinment" and "Target 5-3".

*5.13) SomamiR DB 2.0 (*[*http://compbio.uthsc.edu/SomamiR*](http://compbio.uthsc.edu/SomamiR)*) (46)*

The SomamiR is a comprehensive repository of cancer somatic mutations altering microRNA-ceRNA interactions. We downloaded “miRNA_somatic_v2.0.txt.tar.gz” from SomamiR website (<http://compbio.uthsc.edu/SomamiR/>, Jan., 2018). Furthermore, we extracted miRNA-target relations from microRNA.org, miR IDs were used to map somatic mutations altering miRNA-target relations. Columns including "Mutation", "Mutation ID", "miR ID", "Mutation Seq", "Chr", "Strand", "Sample Name" and "Cancer Type" were retained.

*5.14) miRcode (*[*http://mircode.org/*](http://mircode.org/)*) (47)*

We downloaded both "mircode_highconsfamilies.txt.gz” (~15.20 MB) and “mircode_medconsfamilies.txt.gz” from its website (<http://mircode.org/download.php>, Dec., 2017). Ensembl gene IDs were used to map data from miRcode to iEKPD. We extracted information including "Conservation", "microRNA", "Seed Pos", "Seed Type", "Total Cons %" and "tr Region".

*5.15) RAID v2.0 (*[*http://www.rna-society.org/raid/*](http://www.rna-society.org/raid/)*) (48)*

RAID provided an updated resource for RNA-associated interactions. We downloaded "raid.v2_all_data.zip” from download page of RAID (<http://www.rna-society.org/raid/download.html>, Jan, 2018). Entrez gene IDs were used to map data to iEKPD. Columns entitled "RAID id", "Interactor2", "Category2", "ID2", "Methods", "Databases Source", "References" and "Score" were integrated.

*5.16) LncRNADisease (*[*http://www.cuilab.cn/lncrnadisease*](http://www.cuilab.cn/lncrnadisease)*) (49)*

LncRNADisease was lncRNA and disease association database. We downloaded “exp_lnc_interaction.txt” from LncRNADisease website (<http://www.cuilab.cn/lncrnadisease>, Jan., 2018). Gene names were used as primary key to acquire lncRNA targeting genes in iEKPD. Columns including "lnc RNA", "Interaction", "Ref Seq" and "References" were integrated.

*5.17) OverGeneDB (http://overgenedb.amu.edu.pl) (50)*

The OverGeneDB is a comprehensive repository of 5' end protein coding overlapping genes. We downloaded “overgene.txt” from OverGeneDB website. Gene symbols were used to map data to iEKPD. We integrated information including "Gene 2", "Genes Overlap In:", "Both Genes Expr. In:", "Gene 1 Expr. In:" and "Gene 2 Expr. In:".

*5.18) SEA (**<http://sea.edbc.org/>) (51)*

We downloaded both "humansuperenhancerSEA00101.bed” and “mouseSEA00201.bed” from its website (Jan., 2018). Gene symbols were used to map data from SEA to iEKPD. We extracted information including "SE ID", "Chr", "Start", "End", "Name", "Cell Type", "Mean" and "Median".

**6. DNA Methylation**

*6.1) TCGA (*[*https://cancergenome.nih.gov/*](https://cancergenome.nih.gov/)*) (1)*

We downloaded all available gene methylation profiles for 36 cancers including ACC, BLCA, BRCA, CESC, CHOL, COAD, DLBC, ESCA, GBM, GBMLGG, HNSC, KICH, KIPAN, KIRC, KIRP, LAML, LGG, LIHC, LUAD, LUSC, MESO, OV, PAAD, PCPG, PRAD, READ, SARC, SKCM, STAD, STES, TGCT, THCA, THYM, UCEC, UCS and UVM from BROAD Institute (level3, Jan., 2018). We used Gene symbols to map data to iEKPD. Columns entitled "Project", "Probe", "Sample", "Value" were integrated.

*6.2) ICGC (*[*https://icgc.org/*](https://icgc.org/)*) (2)*

We downloaded all methylation profiles in available ICGC projects including BLCA-US, GBM-US, BRCA-US, CESC-US, CLLE-ES, COAD-US, HNSC-US, KIRC-US, KIRP-US, LAML-US, LGG-US, LIHC-US, LUAD-US, LUSC-US, OV-AU, OV-US, PAAD-US, PACA-AU, PAEN-AU, PBCA-DE, PRAD-CA, PRAD-US, READ-US, SKCM-US, STAD-US, THCA-US and UCEC-US (Jan., 2018). Methylation probe IDs of Infinium HumanMethylation450K and HumanMethylation27 for each gene in iEKPD were retrieved from Illumina (<https://support.illumina.com/array/kits.html>). Columns including "Probe", "Value", "Value type" and "Project" were integrated.

*6.3) COSMIC (*[*https://cancer.sanger.ac.uk/cosmic/download*](https://cancer.sanger.ac.uk/cosmic/download)*) (3)*

We downloaded “CosmicCompleteDifferentialMethylation.tsv.gz” from COSMIC website (<https://cancer.sanger.ac.uk/cosmic/download>, v83). We used Ensembl transcript IDs to map data to iEKPD. Columns including "FRAGMENT_ID", "POSITION", "STRAND", "METHYLATION", "AVG_BETA_VALUE_NORMAL", "BETA_VALUE" and "TISSUE" were integrated.

*6.4) MethyCancer (*[*http://methycancer.psych.ac.cn/*](http://methycancer.psych.ac.cn/)*) (52)*

MethyCancer hosted both data of DNA methylation, cancer-related gene, mutation. We downloaded “MethyCancer_gene_data.txt” and “MethyCancer_gene_cancer_data.txt” from FTP server of MethyCancer (<http://methycancer.psych.ac.cn/Download.do>, Jan, 2018). Ensembl Gene IDs were used to map data to iEKPD. Columns entitled "gene_ID", "gene_cancer_ID", "cancer_ID" and "Tissue" were integrated.

**7. Molecular Interaction**

*7.1) HINT (*[*http://hint.yulab.org/*](http://hint.yulab.org/)*) (53)*

We downloaded all PPI files from HINT website (<http://hint.yulab.org/download/>, Jan., 2018). UniProt IDs were adopted as primary keys to map data to iEKPD. We retained information including "UniProt", "Gene_name", "pmids", "method" and "quality".

*7.2) PINA (*[*http://omics.bjcancer.org/pina/*](http://omics.bjcancer.org/pina/)*) (54)*

First, we downloaded PPI files for 7 model species, including *arabidopsis thaliana*, *caenorhabditis elegans*, *drosophila melanogaster*, *homo sapiens*, *mus musculus*, *rattus norvegicus* and *saccharomyces cerevisiae* (“Arabidopsis thaliana-20140521.tsv”, “Caenorhabditis elegans-20140521.tsv”, “Drosophila melanogaster-20140521.tsv”, “Homo sapiens-20140521.tsv”, “Mus musculus-20140521.tsv”, “Rattus norvegicus-20140521.tsv”, “Saccharomyces cerevisiae-20140521.tsv”), from PINA website (<http://omics.bjcancer.org/pina/interactome.stat.do>, Feb., 2018). UniProt IDs were used to map PPI pairs to iEKPD. Columns entitled "gene_name", "method", "publications", "taxa_B", "interaction_type", "source_DB", "interaction_ID", "Experimental role(s) interactor A" and "Experimental role(s) interactor B" were integrated.

*7.3) Mentha (*[*http://mentha.uniroma2.it/*](http://mentha.uniroma2.it/)*) (55)*

We downloaded file “all.zip” from Mentha website (<http://mentha.uniroma2.it/download.php>, Jan., 2018, version 2018-01-29). UniProt IDs were adopted as primary keys to map data to iEKPD. We retained columns including "UP_ID", "Gene_Name", "Taxa_ID", "Score", "PMIDs".

*7.4) InWeb_IM (*[*https://www.intomics.com/inbio/map.html*](https://www.intomics.com/inbio/map.html)*) (56)*

First, we downloaded “InBio_Map_core_2016_09_12.tar.gz” from download page of InWeb_IM ([https://www.intomics.com/inbio/map.html#downloads](https://www.intomics.com/inbio/map.html%23downloads), Feb., 2018). UniProt IDs were adopted as primary keys to map data to iEKPD. We retained columns including "UP_ID", "Gene_Name", "Taxa_ID", "MI_ID1", "Interaction1", "MI_ID2", "Interaction2" and "Confident score".

*7.5) MIST (*[*http://fgrtools.hms.harvard.edu/MIST/*](http://fgrtools.hms.harvard.edu/MIST/)*) (57)*

We downloaded the “MIST_interaction_genetic_interolog_vs3.zip”, “MIST_interaction_genetic_vs3.zip”, “MIST_interaction_ppi_interolog_vs3.zip” and “MIST_ interaction_ppi_vs3.zip” from download page of MIST (http://fgrtools.hms.harvard.edu /MIST/downloads.jsp, Jan., 2018). We used Entrez IDs as primary keys to map data from MIST to iEKPD. Columns entitled "MasterNetID", "GeneB", "Rank", "Interaction Type", "Exp Direct", "Exp Indirect", "TaxID Interolog", "References" and "Source Interolog" were integrated.

*7.6) IID (*[*http://iid.ophid.utoronto.ca/SearchPPIs/protein/*](http://iid.ophid.utoronto.ca/SearchPPIs/protein/)*) (58)*

All PPI files in IID database were downloaded from IID website (<http://iid.ophid.utoronto.ca/iid/Download/>, Jan., 2018), including “iid.fly.2017-04.txt”, “iid.human.2017-04.txt”, “iid.mouse.2017-04.txt”, “iid.rat.2017-04.txt”, “iid.worm.2017-04.txt”, “iid.yeast.2017-04.txt”. UniProt IDs were used to map data from IID to iEKPD. Information including "symbol1", "pmids", "dbs", "evidence type" and PPI evidence in tissues including "adipose tissue", "adrenal gland", "amygdala", "bone", "bone marrow", "brain", "dorsal root ganglia", "eye", "heart", "hindgut", "hypothalamus", "kidney", "liver", "lung", "lymph nodes", " mammary gland", "midgut", "neurons", "ovary", "pancreas", "pituitary gland", "placenta", "prostate", "salivary gland", "skeletal muscle", "small intestine", "spleen", "stomach", "testes", "uterus" were retained for integration.

*7.7) iRefIndex (*[*http://irefindex.org/wiki/index.php?title=iRefIndex*](http://irefindex.org/wiki/index.php?title=iRefIndex)*) (59)*

We downloaded “All.mitab.04072015.txt.zip” from iRefIndex website (http://irefindex.org /download/irefindex/data/archive/release_14.0/psi_mitab/MITAB2.6/, Dec., 2017, release 14). UniProt IDs were used to map PPI pair to iEKPD. Columns including "B_ID", "method", "pmids", "A_species", "interactionType", "edgetype" and "numParticipants" were integrated.

*7.8) RISE (*[*http://rise.life.tsinghua.edu.cn/*](http://rise.life.tsinghua.edu.cn/)*) (60)*

RISE is a comprehensive and useful repository of RNA-RNA interactions. We downloaded “rise_all.txt.gz” from RISE website (<http://rise.life.tsinghua.edu.cn/downloads.html>, Apr., 2018). Ensembl gene IDs were used to map data to iEKPD. Columns including "Chr2", "Start2", "End2", "Rise ID", "Score", "Strand2", "Gene ID2", "Gene Name2", "Method" and "References" were extracted and integrated.

*7.9) DifferentialNET (*[*http://netbio.bgu.ac.il/diffnet/*](http://netbio.bgu.ac.il/diffnet/)*) (61)*

We downloaded PPI file for each tissue from download page of DifferentialNet (<http://netbio.bgu.ac.il/labwebsite/?q=differentialnet-download>, Feb., 2018). Ensembl Gene IDs were used to map data to iEKPD. Columns including "Interacting Gene", "Interact Type" and "Tissue" were retained and integrated.

*7.10) TRRUST (*[*http://www.grnpedia.org/trrust/*](http://www.grnpedia.org/trrust/)*) (62)*

TRRUST is an expanded repository of transcriptional regulatory interactions focusing on human and mouse transcriptional regulatory networks. “trrust_rawdata.human.tsv” and “trrust_rawdata.mouse.tsv” from download page of TRRUST (http://www.grnpedia.org /trrust/downloadnetwork.php, Jan., 2018) were downloaded. Ensembl gene IDs were used as primary keys to map data from TRRUST to iEKPD. Columns entitled "Direction", "Partner", "Relationship" and "References" were integrated.

*7.11) TIMBAL (*[*http://mordred.bioc.cam.ac.uk/timbal*](http://mordred.bioc.cam.ac.uk/timbal)*) (63)*

We downloaded “TIMBAL_sm.csv” from TIMBAL website (http://mordred.bioc.cam.ac.uk /timbal/all, Jan., 2018). UniProt IDs were used to map PPI pair to iEKPD. Columns including "Target Name", "Literature Name", "B Complex Description", "PDB Code", "Activity", "Assay Type" and "References" were integrated.

*7.12) BindingDB (*[*http://www.bindingdb.org/bind/index.jsp*](http://www.bindingdb.org/bind/index.jsp)*) (64)*

BindingDB comprised about 1,454,892 binding data. We downloaded “BindingDB_All_2018m4.tsv.zip” from BindingDB website (https://www.bindingdb.org /bind/chemsearch/marvin/SDFdownload.jsp?all_download=yes, Jan., 2016). UniProt IDs were used to map data to iEKPD. Columns including "BindingDB Reactant Set ID", "Ki (nM)", "IC50 (nM)", "Kd (nM)", "EC50 (nM)", "kon (M-1-s-1)", "koff (s-1)", "pH", "Temp (C)", "Drugbank ID" and "References" were integrated.

*7.13) PLIC (*[*http://proline.biochem.iisc.ernet.in/PLIC/index.php*](http://proline.biochem.iisc.ernet.in/PLIC/index.php)*) (65)*

We downloaded the "Atomic_contacts.zip”, "Binding_Site_descriptors.zip”, "Database_binding_sites_information.zip”, "Experimental_binding_affinity.zip” and "Residue_Contacts.zip” from PLIC download page (http://proline.biochem.iisc.ernet.in /PLIC/download.php#, Apr., 2014). UniProt IDs were used as primary keys to map data from PLIC to iEKPD. Columns entitled "Binding Site ID", "Binding Site Name", "PDB ID", "Chain ID", "Ligand ID", "Ligand Name" and "Cath Supfam ID" were retained and integrated.

*7.14) RAIN (*[*http://rth.dk/resources/rain*](http://rth.dk/resources/rain)*) (66)*

RAIN provided both ncRNA-RNA and ncRNA-protein interactions. "v1.database.tsv.gz”, "v1.experiments.tsv.gz”, "v1.predictions.tsv.gz” and "v1.textmining.tsv.gz” were downloaded from its download page (<https://rth.dk/resources/rain/download.html>, Jan., 2018). We took Ensembl protein IDs as primary keys to map the relationship to iEPKD. We integrated information including "ncRNA", "Type", "Confidence", "Source" and "References".

*7.15) YTRP (*[*http://cosbi3.ee.ncku.edu.tw/YTRP/*](http://cosbi3.ee.ncku.edu.tw/YTRP/)*) (67)*

We downloaded the “TRP_binding_network.txt” and “TRP_direct_regulatory_network.txt” from YTRP (<http://cosbi3.ee.ncku.edu.tw/YTRP/Download>, Mar., 2018). Then, we used gene symbols as primary keys to map data from YTRP to iEKPD. Columns entitled "TF", "Pathway" and "Network" were integrated.

*7.16) RegNetwork (*[*http://www.regnetworkweb.org/*](http://www.regnetworkweb.org/)*) (68)*

We downloaded the “human.zip” and “mouse.zip” from RegNetwork (<http://www.regnetworkweb.org/download.jsp>, Jan., 2018). Then, we used Gene symbols as primary keys to map data from RegNetwork to iEKPD. We integrated information including "Protein ID", "Target", "ID" and "Up or Down or Unknown".

**8. Drug-target relation**

*8.1) TTD (*[*http://bidd.nus.edu.sg/group/cjttd/*](http://bidd.nus.edu.sg/group/cjttd/)*) (69)*

First, we downloaded TTD targets information in raw format (“P1-01-TTD_download.txt”) from download page of TTD website (<https://db.idrblab.org/ttd/full-data-download>, version 6.1.01). UniProt IDs were used as primary keys to find drugs targeting proteins in iEKPD. Drug names, type of targets, drug synonyms and associated disease were extracted and integrated.

*8.2) DrugBank (*[*https://www.drugbank.ca/*](https://www.drugbank.ca/)*) (70)*

*We downloaded* *“drugbank_all_full_database.xml.zip” from download page of DrugBank* (<https://www.drugbank.ca/releases/latest>, version 2017-11-06). UniProt IDs were used as primary keys to find drugs targeting proteins in iEKPD. “DrugBank ID”, “drug names”, “groups”, “know-action” and “PMIDs” were extracted and integrated.

*8.3) ADReCS-Target (*[*http://bioinf.xmu.edu.cn/ADReCS-Target/*](http://bioinf.xmu.edu.cn/ADReCS-Target/)*) (71)*

First, we downloaded “P_D_A.xlsx” from ADReCS-Target (<http://bioinf.xmu.edu.cn/ADReCS-Target/download.jsp>, Jan., 2018). UniProt IDs were used as primary keys to find drugs targeting proteins in iEKPD. Columns including "BADD_TID", "ADR_ID", "ADReCS ID", "ADR Term", "Uniprot AC" and "Drug_Name" were integrated.

*8.4) ECOdrug (*[*http://www.ecodrug.org/*](http://www.ecodrug.org/)*) (72)*

We downloaded “ECOdrug_ensembl.csv” from ECOdrug (http://www.ecodrug.org /#downloads, Jan., 2018). We used Ensembl gene IDs as primary keys to map data from ECOdrug to iEKPD. Columns entitled "Drug_name", "MoA_text", "DrugbankID", "Drug_type", "Drug_FirstApproval", "ATC.code", "Target_ChEMBLID", "Target_pref_name", "Target_name", "Target_class" and "interaction" were integrated.

*8.5) DGIdb 3.0 (*[*http://www.dgidb.org/*](http://www.dgidb.org/)*) (73)*

We downloaded “interactions.tsv” from DGIdb (<http://www.dgidb.org/downloads>, Jan., 2018). We took Entrez ID as primary keys to map drug-target relationship to iEPKD. We integrated information including "interaction_claim_source", "interaction_types", "drug_claim_name", "drug_claim_primary_name", "drug_name" and "drug_chembl_id".

*8.6) KPID (*[*http://www.kinase-screen.mrc.ac.uk*](http://www.kinase-screen.mrc.ac.uk)*)*

We retrieved all inhibitors for all available genes in KPID (“kinaseinhibitorNEW.txt”, Jan., 2018). UniProt IDs were used to map data to iEKPD. Columns including "Inhibitor", "Brutto", "MW", "Target" and "Reference" were included.

*8.7) GRAC (*[*http://www.guidetopharmacology.org/targets.jsp*](http://www.guidetopharmacology.org/targets.jsp)*) (74)*

We downloaded “interactions.txt” from GRAC (<http://www.guidetopharmacology.org/download.jsp>, Jan., 2018). UniProt IDs were used as primary keys to find drugs targeting proteins in iEKPD. Columns including "ligand", "ligand id", "lignad pubchem id", "type", "action", "affinity units", "affinity" and "reference" were integrated.

*8.8) PDTD (*[*http://dddc.ac.cn/pdtd*](http://dddc.ac.cn/pdtd)*) (75)*

The PDTD website cannot be accessed for now, we used a previously downloaded dataset to map data to iEKPD (“allSiteFiles.tar.gz”, Feb., 2018). UniProt IDs were used to map data to iEKPD, we integrated "UniProt ID" and "PDB code" for each match.

*8.9) CTD (*[*http://ctdbase.org/*](http://ctdbase.org/)*) (76)*

First, we downloaded “CTD_chem_gene_ixns.tsv.gz” from CTD (<http://ctdbase.org/downloads/>, Feb, 2018). Entrez IDs were used to retrieve drug-target relationships from CTD. Columns entitled "ChemicalName", "ChemicalID", "CasRN", "GeneSymbol", "InteractionActions" and PubMedIDs" were integrated.

**9. Protein 3D Structure**

*9.1) PDB (*[*http://www.rcsb.org/*](http://www.rcsb.org/)*) (77)*

We downloaded all PDB files from FTP site of PDB ([ftp://ftp.wwpdb.org/pub/pdb/data /structures/all/pdb/](ftp://ftp.wwpdb.org/pub/pdb/data%20/structures/all/pdb/)). UniProt IDs were used to map data to iEKPD. Columns including "PDB ID", "Chain", "Molecule" and "Fragment" were integrated.

*9.2) MMDB (*[*https://www.ncbi.nlm.nih.gov/structure/*](https://www.ncbi.nlm.nih.gov/structure/)*) (78)*

We downloaded the “nrpdb..gz” from FTP server of MMDB (ftp://ftp.ncbi.nih.gov /mmdb/nrtable/, March, 2018). We firstly extracted all PDB code together with corresponding UniProt ID from PDB, then we use PDB code as primary key to map MMDB data to iEKPD. The column entitled “PDB code” (1), “Chain ID” (2), “MMDB ID” (3), “Resolution” (K), “Method of coordinate determination” (P), “Acceptable in structural quality” (Q) were retained for integration.

*9.3) SCOP (*[*http://scop2.mrc-lmb.cam.ac.uk/*](http://scop2.mrc-lmb.cam.ac.uk/)*) (79)*

We downloaded the “scop2_nodes_names_20140205”, “domain_segments_pdb_ 20140205”, “domain_segments_seq_20140205”, “domains2nodes_20140205” from download page of SCOP2 (<http://scop2.mrc-lmb.cam.ac.uk/downloads/>, Feb., 2018). We obtained domain IDs and locations on proteins for entries in iEKPD from “domain_segments_seq_20140205”. For mapped entries, we further extracted PDB codes and exact position of SCOP domains from “domain_segments_pdb_20140205”. SCOP node IDs for all domains were took from “domains2nodes_20140205”. Furthermore, we extracted names for mapped nodes from “scop2_nodes_names_20140205”. Finally, the columns entitled "Domain", "Domain_serial", "Node", "Name", "PDB", "Chain", "BeginPDB", "EndPDB", "UniprotKB", "BeginUni", "EndUni" were retained for integration.

**10. Post-translational Modification**

*10.1) PLMD (*[*http://plmd.biocuckoo.org/*](http://plmd.biocuckoo.org/)*) (80)*

We downloaded the “Total.zip” from (<http://plmd.biocuckoo.org/download.php>, Jan., 2018). UniProt IDs were used to map data from PLMD to iEKPD. Columns entitled "PLMD Acc", "Position", "Code", "Type" and "References" were retained for integration.

*10.2) dbPAF (*[*http://dbpaf.biocuckoo.org/*](http://dbpaf.biocuckoo.org/)*) (81)*

We downloaded the “Total.zip” from (<http://dbpaf.biocuckoo.org/download.php>, Jan., 2018). UniProt IDs were used to map data from dbPAF to iEKPD. Columns entitled "dbPAF ID", "Position", "Type" and "References" were retained for integration.

*10.3) dbPPT (*[*http://dbppt.biocuckoo.org/*](http://dbppt.biocuckoo.org/)*) (82)*

We downloaded the “data.txt” from (<http://dbppt.biocuckoo.org/download.php>, Jan., 2018). UniProt IDs were used to map data from dbPPT to iEKPD. Finally, the columns entitled "Position", "Peptide", "Theoretical PI", "Molecule Weight" and "References" were retained for integration.

*10.4) PhosSNP (*[*http://phossnp.biocuckoo.org/*](http://phossnp.biocuckoo.org/)*) (83)*

We downloaded the “phossnp.txt” from (<http://phossnp.biocuckoo.org/down.php>, Dec., 2017). UniProt IDs were used to map data from PhosSNP to iEKPD. Finally, the columns entitled "Substrate Family", "TK Writers", "SH2/PTB Readers", "Dual-/Singular-Role Substrates", "Signaling Routes", "Tissue-Specificity", "Evo-Patterns" and "Number of Samples" were retained for integration.

*10.5) PhosphoSitePlus (*[*http://www.phosphosite.org/*](http://www.phosphosite.org/)*) (84)*

We downloaded 14 files including "Acetylation_site_dataset.gz”, "Methylation_site_dataset.gz”, "O-GalNAc_site_dataset.gz”, "O-GlcNAc_site_dataset.gz”, "Phosphorylation_site_dataset.gz”, "Sumoylation_site_dataset.gz” and "Ubiquitination_site_ dataset.gz” from download page of PhosphoSitePlus (https://www.phosphosite.org /staticDownloads, Jan., 2018). UniProt IDs were used to map data from PhosphoSitePlus to iEKPD. We integrated information including "Gene", "Protein", "Position", "MOD RSD", "Site Grp ID" and "Peptide".

*10.6) dbPTM (*[*http://dbptm.mbc.nctu.edu.tw/index.php*](http://dbptm.mbc.nctu.edu.tw/index.php)*) (85)*

dbPTM was designed to be knowledgebase of protein post-translational modifications (PTMs). We downloaded “dbPTM3.txt” from download page of dbPTM (<http://dbptm.mbc.nctu.edu.tw/download.php>, Jan., 2018). UniProt IDs were used to map data from dbPTM to iEKPD. Columns entitled 'UniProt ID", "Position", "Code", "Type", "Resource" and "References" were retained for integration.

*10.7) HPRD (*[*http://www.hprd.org/*](http://www.hprd.org/)*) (86)*

We download “POST_TRANSLATIONAL_MODIFICATIONS.txt” from its download page (<http://hprd.org/download>, Feb., 2018). We use RefSeq protein IDs as primary keys to map data from HPRD to iEKPD. Finally, we integrated information including "NP ID", "Position", "Code", "Type", "Resource" and "References".

*10.8) Phospho.ELM (*[*http://phospho.elm.eu.org/*](http://phospho.elm.eu.org/)*) (87)*

We downloaded “phosphoELM.txt” from Phospho.ELM (April, 2018). UniProt IDs were used to map data from Phospho.ELM to iEKPD. Finally, the columns entitled "Acc", "Position", "Code", "Kinases" and "References" were retained for integration.

*10.9) UniProt (*[*http://www.uniprot.org/*](http://www.uniprot.org/)*) (88)*

We downloaded “uniprot_sprot.dat” from UniProt (April, 2018). UniProt IDs were used to map data from UniProt to iEKPD. All PTM sites except for those annotated with “By similarity”, “Potential” or “Probable” were considered for further integration. Finally, the columns entitled "UniProt ID", "Position", "Type" and "References" were integrated.

*10.10) PHOSIDA (*[*http://141.61.102.18/phosida/index.aspx*](http://141.61.102.18/phosida/index.aspx)*) (89)*

We downloaded all available data for 6 species including *C. elegans, D. melanogaster, S. cerevisiae, H. sapiens, M. musculus and H. salinarium* (May, 2018)*.* As PHOSIDA used different IDs for different species, Ensembl gene IDs were used as primary keys for *C. elegans, D. melanogaster* and *S. cerevisiae,* gene symbols were used for *H. sapiens* and *M. musculus,* while UniProt IDs were used for *H. salinarium*. Columns entitled "Description", "Amino Acid", "Position", "Peptide Sequence" and "Surrounding References" were integrated.

*10.11) BioGRID (*[*https://thebiogrid.org/*](https://thebiogrid.org/)*) (90)*

The Biological General Repository for Interaction Datasets (BioGRID) is an open access database which contains 38 559 post-translational modifications. We downloaded “BIOGRID-PTM-3.4.159.ptmtab.txt” from download page of BioGRID (<http://dbptm.mbc.nctu.edu.tw/download.php>, Jan., 2018) and used Entrez IDs to map data from BioGRID to iEKPD. Finally, the columns entitled "BioGRID ID", "Position", "Code", "Modification", "Organism", "Source Database" and "References" were retained for integration.

*10.12) O-GlycBase (*[*http://www.cbs.dtu.dk/databases/OGLYCBASE/*](http://www.cbs.dtu.dk/databases/OGLYCBASE/)*) (91)*

We downloaded “Oglyc.base.txt” from O-GlycBase (Jan., 2018) and used UniProt IDs to map data from O-GlycBase to iEKPD. Finally, the columns entitled "DB ID", "SER Sites", "THR Sites", "ASN Sites", "TRP Sites" and "References" were retained for integration.

*10.13) PhosphoBase (*[*http://www.cbs.dtu.dk/databases/PhosphoBase/pbase2/*](http://www.cbs.dtu.dk/databases/PhosphoBase/pbase2/)*) (92)*

We downloaded “phosphobase.dat” from PhosphoBase (May, 2018) and used UniProt IDs to map data to iEKPD. Columns entitled "DB ID", "UP ID", "S Sites", "T Sites", "Y Sites" were retained for integration.

*10.14) mUbiSiDa (*[*http://202.195.183.4:8000/mUbiSiDa.php*](http://202.195.183.4:8000/mUbiSiDa.php)*) (93)*

mUbiSida deposited experimentally validated 110,976 ubiquitination sites in 35,494 ubiquitinated proteins. We downloaded “data_2013_10_12.rar” from download page of mUbiSiDa (<http://202.195.183.4:8000/resources.php>, April, 2018) and used UniProt IDs to map data to iEKPD. Finally, we integrated information including "UniProt ID", "Ubi Site", "Ubi Seq", "Organism" and "References".

**11. Protein Expression/Proteomics**

*11.1) The Human Protein Atlas (*[*http://www.proteinatlas.org/*](http://www.proteinatlas.org/)*) (27)*

The Human Protein Atlas provided various downloadable data. We downloaded the “subcellular_location.tsv” and “proteinatlas.xml” from download page (https://www.proteinatlas.org /about/download, Jan., 2018). UniProt IDs as primary keys to map data. Columns entitled "Synonyms", "Antibody IDs", "Reliability", "Location", "Average (tissue)" and "Average (celline)" were integrated.

*11.2) Human Proteome Map (*[*http://www.humanproteomemap.org/*](http://www.humanproteomemap.org/)*) (28)*

We downloaded the “HPM_peptide_genes_Kim_et_al_052914.xlsx”, “HPM_peptide_level_ expression_matrix_Kim_et_al_052914.xlsx”, from download page (http://www.human proteomemap.org/download.php, April, 2018). We used RefSeq protein IDs to map peptide to iEKPD, while expression level of each protein in all tissues were extracted from “HPM_peptide_level_expression_matrix_Kim_et_al_052914.xlsx”. Finally, the columns entitled "Peptide", "Adult Adrenal Gland", "Adult Colon", "Adult Esophagus", "Adult Frontal Cortex", "Adult Gallbladder", "Adult Heart", "Adult Kidney", "Adult Liver", "Adult Lung", "Adult Ovary", "Adult Pancreas", "Adult Prostate", "Adult Rectum", "Adult Retina", "Adult Spinal Cord", "Adult Testis", "Adult Urinary Bladder", "Fetal Brain", "Fetal Gut", "Fetal Heart", "Fetal Liver", "Fetal Ovary", "Placenta", "Fetal testis", "B Cells", "CD4 T Cells", "CD8 T Cells", "Monocytes", "NK Cells" and "Platelets" were retained for integration.

**12. Subcellular Localization**

*12.1) NLSdb (*[*https://rostlab.org/services/nlsdb/*](https://rostlab.org/services/nlsdb/)*) (94)*

All data in NLSdb were stored using CSV files. We downloaded the “nuclear.csv”, “signals.csv” from download page of NLSdb (<https://rostlab.org/services/nlsdb/downloads>, Jan., 2018). UniProt IDs as primary keys to map data from NLSdb to iEKPD. Columns entitled "SignalType", "AnnotationType", "ConfidenceNuc", "ConfidenceFam" and "Origin" were integrated.

*12.2) COMPARTMENTS (*[*https://compartments.jensenlab.org/*](https://compartments.jensenlab.org/)*) (95)*

COMPARTMENTS provided a lot of useful information on protein subcellular localization for researchers. We downloaded known, predicted and text-mining localizations for human, mouse, rat, worm, yeast and *A. thalianana* from download page of COMPARTMENTS (<https://compartments.jensenlab.org/Downloads>, Jan., 2018). Ensembl gene IDs were used to map data from COMPARTMENTS to iEKPD. Finally, we integrated information including "GO term", "Localization", "Source", "Organism", "Type" and "Confidence".

**13. Protein Functional Annotation**

*13.1) CGDB (*[*http://cgdb.biocuckoo.org/*](http://cgdb.biocuckoo.org/)*) (96)*

Previously, we developed a database of circadian genes in eukaryotes, containing ∼73 000 circadian-related genes in 68 animals, 39 plants and 41 fungi (96). We used UniProt IDs to map data from CGDB to iEKPD. Columns entitled "CGDB ID", "Uniprot/Ensembl ID", "Protein Name", "Organism", "Evidence" were retained for integration.

*13.2) THANATOS (*[*http://thanatos.biocuckoo.org*](http://thanatos.biocuckoo.org)*) (97)*

Recently, we developed a comprehensive database of The Autophagy, Necrosis, ApopTosis OrchestratorS (THANATOS), containing 191,543 proteins potentially associated with autophagy and cell death pathways in 164 eukaryotes (97). Here, we used UniProt IDs to map data to iEKPD. Columns including "UniProt ID", "Pathway", "Function" and "Reference" were integrated.

*13.3) RaftProt (*[*http://lipid-raft-database.di.uq.edu.au/*](http://lipid-raft-database.di.uq.edu.au/)*) (98)*

We retrieved all the mammalian lipid raft-associated proteins which come from six files such as “Bovine.csv”, “Hamster.csv”, “Human.csv”, “Monkey.csv”, “Mouse.csv” and “Rat.csv” in RaftProt. We used UniProt IDs to map data to iEKPD. We integrated information including "Entry Name", "Confidence", "Experiment Count", "Gene Confidence" and "Other Gene Names".

**Supplementary Results**

**The classification of known phospho-regulators**

In EKPD 1.0, we classified curated PKs into 10 groups with 148 families (99). Here, PKs were re-classified into 151 families of 11 groups (Figure 2). First, the FAM20C family in the aPK group was changed into the FJ (Four-jointed) family of a new group named PKL (Protein Kinase-Like) based on the update of Kinase.com (100). Also, we added four new families under the aPK group as PKM2, PGK1, KHK-A and HK for the metabolic kinases that can act as serine/threonine PKs (101). The HisK family was not reserved any longer because iEKPD 2.0 focused on the collection and annotation of phospho-regulators for pS/pT/pY residues.

Previously, we classified known PPs into 10 groups with 33 families (99). In iEKPD 2.0, PPs were re-classified into 36 families of 13 groups (Figure 2). Three new groups were added, including the His-Based PSP group (102), the His-Based PTP group (102) and the ASP-Based PSP group (103). The PGAM5 family was added in the His-Based PSP group given that human PGAM5 can dephosphorylate pS/pT residues in MAP3K5 (102,104). Two families of TULA-1 and TULA-2 in the PTP-Other group were merged into a single family as TULA based on sequence similarity and re-classified into the His-Based PTP group, which also contained an additional family of ACP for ACP4/ACPT and ACPP (102,105). As previously described (103), two mistakenly classified families of CIN (Chronophin) and FCP (TFIIF-associating component of C-terminal domain phosphatase) of the Asp-Based PTP in EPKD 1.0 were re-classified into the ASP-Based PSP group. In addition, we added PGP (Phosphoglycolate phosphatase) family and AP (Alkaline phosphatase) family to the Asp-Based PTP group and PTP-Other group, respectively (106). Taken together, PKs were classified into 151 families of 11 groups, whereas PPs were classified into 36 families of 13 groups (Figure 2).

In 2017, Chen *et al.* developed the Phosphatome.net database and integrated 1,425 known and predicted PP genes in nine eukaryotes. These PPs were then structurally classified into 10 protein folds (groups), 21 families, and 178 subfamilies, based on the SCOP database *(79).* By comparison of the PP classifications in iEKPD 2.0 and Phosphatome.net, we found that 8 families including CDC25, LMWPTP, EYA, FCP, PTEN, Myotubularin, SUS72 and AP in our database were exactly identical to Phosphatome.net (106). Five families including PHP, Sac, Paladin, OCA and PAP in Phosphatome.net were not considered, because no pS/pT/pY residues in protein substrates were experimentally identified to be modified by members in these families. Four original families in Phosphatome.net including PTP, DSP, PPP and PPM were regarded as groups according to the functional diversity of members in these families (111,113). As previously described (111), the original PTP family was changed to the PTP/Classical PTP group, which contained NRPTP and RPTP families, in iEKPD 2.0. The DSP family in Phosphatome.net was changed to the PTP/DSP group, which contained 7 families including aDSP, MKP, PRL, CDC14, SSH, Myotubularins and PTEN (111). Also, the original PPP family was changed to PSP/PPP group containing 8 families such as PP1, PP2A, PP2B, PP4, PP5, PP6, PP7, Kelch, SLP and Unique, whereas the original PPM family was changed to the PSP/PPM group, which contained PP2C and PDC families (111,113). Again, due to the functional diversity, the HP1 family in Phosphatome.net was divided into PSP/His-Based PSP/PGAM5 and PTP/His-Based PTP/TULA families (102). Similarly, the original NagD family was divided into PSP/Asp-Based PSP/CIN and PTP/Asp-Based PTP/PGP families (112). Moreover, the original HP2 family was renamed as the ACP family and only members that dephosphorylate pS/pT/pY residues were reserved (102,105). The original RTR1 family was renamed as the RPAP2 family, because the latter is the standard gene name. In addition, three new families were added, including PSP/PSP-Other/Caspy_SIP (107,108), PSP/PPP/PPP_Unique (109) and PTP/Asp-Based PTP/MDP1 (110).

The classification of PPBD-containing proteins is relatively simpler and more direct because different types of PPBDs have distinct structures with low sequence similarity. According to previous studies (114-121), we first classified PPBDs into the pS/pT group and the pY group. For the pS/pT group, 14 families were established, including 14-3-3, BRCT (BRCA1 carboxyl-terminal), FHA (forkhead-associated), MH2 (Mad homology 2), PBD, WW, WD40, KIX (kinase-inducible domain interacting domain), IRF3 (Interferon-regulatory factor 3), GK (Guanylate kinase) (122), Arrestin (123), FF (two conserved Phe residues containing domain), PDZ (Domain present in PSD-95, Dlg, and ZO-1/2) and LRR (leucine-rich repeat). For the pY group, 7 families were added, including SH2 (Src homology 2), PTB (phosphotyrosine-binding), C2 (Protein kinase C conserved region 2), PH (pleckstrin homology), HYB (Hakai phospho-tyrosine binding domain), CBL_PTB (Cbl-type phosphotyrosine-binding) and Other (unclassified proteins) (Figure 2).

**The methodology of HMM-based predictions**

Using 176 HMM profiles, we first adopted the hmmsearch program to score all protein sequences in 164 eukaryotes, under the default threshold (E-value = 10). To reduce false positive hits, we further detected potential orthologs of all known PKs and PPs in the remaining 163 species, by using the reciprocal best hits (RBH) approach ([40](#_ENREF_40)). The functional domains in these orthologs were verified from Pfam ([33](#_ENREF_33)), InterPro ([34](#_ENREF_34)) and UniProt ([35](#_ENREF_35)) to ensure these proteins to be potential PKs and PPs. Although hmmsearch calculated both E-values and log-odds likelihood scores for given protein sequences, we chose log-odds likelihood scores as the thresholds, because E-values are not constant and depend on the database size. Then for each PK and PP family, a log-odds score was manually selected as the cut-off value to promise all curated proteins and human orthologs to be included. Because a number of PKs and PPs might be predicted as members of multiple families with different scores, here we determined their classifications based on the highest log-odds scores to ensure that one PK or PP was only assigned into a single family. For each PPBD family, a log-odds threshold value was selected to allow all curated proteins to be reserved. One PPBD-containing protein can be assigned into multiple families based on the family signature domains. For the construction of iEKPD 2.0, the manual classifications of known phospho-regulators were not changed, whereas predicted proteins were computationally classified.

**The performance evaluation of HMM identifications**

The performance tests were separately conducted for PKs, PPs and PPBDs. For PKs, we took known annotated proteins in each family as positive data (*P*), whereas all proteins classified in other families were taken as negative data (*N*). Among the data with positive predictions by hmmsearch, the real positives were defined as *true positives* (*TP*), whereas the others were regarded as *false positives* (*FP*). Among the data with negative predictions by hmmsearch, the real positives were taken as *false negatives* (*FN*), while the others were called as *true negatives* (*TN*). Then four measurements of accuracy (*Ac*), sensitivity (*Sn*), specificity (*Sp*) and Mathew Correlation Coefficient (*MCC*) were adopted to evaluate the prediction performance, and defined as shown below:

$$Ac=\frac{TP+TN}{TP+TN+FP+FN}, Sn=\frac{TP}{TP+FN}, Sp=\frac{TN}{TN+FP}$$

$$MCC=\frac{\left( TP\times TN \right)-(FN\times FP)}{\sqrt{(TP+FN)(TN+FP)(TP+FP)(TN+FN)}}$$

For each PK family, both the self-consistency and leave-one-out (LOO) validations were performed. In the self-consistency validation, the positive and negative proteins in the training data set were directly used to evaluate the performance. However, the computational model might be overfitting without satisfying accuracy for new data, and the LOO validation should be performed for a justified evaluation. In the LOO validation, each protein in the training data set was singled out in turn, whereas all remaining proteins were taken for training a new model to score the protein. The process was repeated until all proteins were used as the independent test samples one time. Under different values of log-odds scores, *Ac*, *Sn*, *Sp* and *MCC* values were computed, respectively. Receiver operating characteristic (ROC) curves were drawn, and area under ROC (AUC) values were calculated. Also, plots of *Ac*, *Sn* and *Sp* values against the different log-odds scores were illustrated. Such a procedure was also adopted for PPs and PPBD-containing proteins. For simplicity, the results of two PK families, one PP family and one PPBD family are presented (Supplementary Figure S1). In addition, the *Ac*, *Sn*, *Sp* and *MCC* values of the self-consistency and LOO validations were calculated for the manually selected log-odds thresholds of 176 families (Supplementary Table S3).

**Supplementary References**

1. Blum, A., Wang, P. and Zenklusen, J.C. (2018) SnapShot: TCGA-Analyzed Tumors. *Cell*, **173**, 530.
2. Joly, Y., Dove, E.S., Knoppers, B.M., Bobrow, M. and Chalmers, D. (2012) Data sharing in the post-genomic world: the experience of the International Cancer Genome Consortium (ICGC) Data Access Compliance Office (DACO). *PLoS Comput. Biol.*, **8**, e1002549.
3. Forbes, S.A., Beare, D., Boutselakis, H., Bamford, S., Bindal, N., Tate, J., Cole, C.G., Ward, S., Dawson, E., Ponting, L. *et al.* (2017) COSMIC: somatic cancer genetics at high-resolution. *Nucleic Acids Res.*, **45**, D777-D783.
4. Krizman, D.B., Wagner, L., Lash, A., Strausberg, R.L. and Emmert-Buck, M.R. (1999) The Cancer Genome Anatomy Project: EST sequencing and the genetics of cancer progression. *Neoplasia*, **1**, 101-106.
5. Gundem, G., Perez-Llamas, C., Jene-Sanz, A., Kedzierska, A., Islam, A., Deu-Pons, J., Furney, S.J. and Lopez-Bigas, N. (2010) IntOGen: integration and data mining of multidimensional oncogenomic data. *Nat. methods*, **7**, 92-93.
6. Dingerdissen, H.M., Torcivia-Rodriguez, J., Hu, Y., Chang, T.C., Mazumder, R. and Kahsay, R. (2018) BioMuta and BioXpress: mutation and expression knowledgebases for cancer biomarker discovery. *Nucleic Acids Res.*, **46**, D1128-D1136.
7. Hu, X., Wang, Q., Tang, M., Barthel, F., Amin, S., Yoshihara, K., Lang, F.M., Martinez-Ledesma, E., Lee, S.H., Zheng, S. *et al.* (2018) TumorFusions: an integrative resource for cancer-associated transcript fusions. *Nucleic Acids Res.*, **46**, D1144-D1149.
8. Sherry, S.T., Ward, M.H., Kholodov, M., Baker, J., Phan, L., Smigielski, E.M. and Sirotkin, K. (2001) dbSNP: the NCBI database of genetic variation. *Nucleic Acids Res.*, **29**, 308-311.
9. Song, S., Tian, D., Li, C., Tang, B., Dong, L., Xiao, J., Bao, Y., Zhao, W., He, H. and Zhang, Z. (2018) Genome Variation Map: a data repository of genome variations in BIG Data Center. *Nucleic Acids Res.*, **46**, D944-D949.
10. Li, J., Shi, L., Zhang, K., Zhang, Y., Hu, S., Zhao, T., Teng, H., Li, X., Jiang, Y., Ji, L. *et al.* (2018) VarCards: an integrated genetic and clinical database for coding variants in the human genome. *Nucleic Acids Res.*, **46**, D1039-D1048.
11. Krassowski, M., Paczkowska, M., Cullion, K., Huang, T., Dzneladze, I., Ouellette, B.F.F., Yamada, J.T., Fradet-Turcotte, A. and Reimand, J. (2018) ActiveDriverDB: human disease mutations and genome variation in post-translational modification sites of proteins. *Nucleic Acids Res.*, **46**, D901-D910.
12. Simonetti, F.L., Tornador, C., Nabau-Moreto, N., Molina-Vila, M.A. and Marino-Buslje, C. (2014) Kin-Driver: a database of driver mutations in protein kinases. *Database (Oxford)*, **2014**, bau104.
13. Zheng, Y., Nie, P., Peng, D., He, Z., Liu, M., Xie, Y., Miao, Y., Zuo, Z. and Ren, J. (2018) m6AVar: a database of functional variants involved in m6A modification. *Nucleic Acids Res.*, **46**, D139-D145.
14. Guo, L. and Wang, J. (2018) rSNPBase 3.0: an updated database of SNP-related regulatory elements, element-gene pairs and SNP-based gene regulatory networks. *Nucleic Acids Res.*, **46**, D1111-D1116.
15. Landrum, M.J., Lee, J.M., Benson, M., Brown, G., Chao, C., Chitipiralla, S., Gu, B., Hart, J., Hoffman, D., Hoover, J. *et al.* (2016) ClinVar: public archive of interpretations of clinically relevant variants. *Nucleic Acids Res.*, **44**, D862-868.
16. Li, M.J., Liu, Z., Wang, P., Wong, M.P., Nelson, M.R., Kocher, J.P., Yeager, M., Sham, P.C., Chanock, S.J., Xia, Z. *et al.* (2016) GWASdb v2: an update database for human genetic variants identified by genome-wide association studies. *Nucleic Acids Res.*, **44**, D869-876.
17. Xu, H., Wang, Y., Lin, S., Deng, W., Peng, D., Cui, Q. and Xue, Y. (2018) PTMD: A Database of Human Disease-associated Post-translational Modifications. *Genomics Proteomics Bioinformatics*.
18. Amberger, J.S., Bocchini, C.A., Schiettecatte, F., Scott, A.F. and Hamosh, A. (2015) OMIM.org: Online Mendelian Inheritance in Man (OMIM(R)), an online catalog of human genes and genetic disorders. *Nucleic Acids Res.*, **43**, D789-798.
19. Yue, M., Zhou, D., Zhi, H., Wang, P., Zhang, Y., Gao, Y., Guo, M., Li, X., Wang, Y., Zhang, Y. *et al.* (2018) MSDD: a manually curated database of experimentally supported associations among miRNAs, SNPs and human diseases. *Nucleic Acids Res.*, **46**, D181-D185.
20. Zhang, G., Shi, J., Zhu, S., Lan, Y., Xu, L., Yuan, H., Liao, G., Liu, X., Zhang, Y., Xiao, Y. *et al.* (2018) DiseaseEnhancer: a resource of human disease-associated enhancer catalog. *Nucleic Acids Res.*, **46**, D78-D84.
21. Lee, K., Lee, S., Park, S., Kim, S., Kim, S., Choi, K., Tan, A.C. and Kang, J. (2016) BRONCO: Biomedical entity Relation ONcology COrpus for extracting gene-variant-disease-drug relations. *Database (Oxford)*, **2016**.
22. Sahajpal, R., Kandoi, G., Dhiman, H., Raj, S., Open Source Drug Discovery, C., Scaria, V., Bhartiya, D. and Hasija, Y. (2014) HGV&TB: a comprehensive online resource on human genes and genetic variants associated with tuberculosis. *Database (Oxford)*, **2014**, bau112.
23. Pinero, J., Bravo, A., Queralt-Rosinach, N., Gutierrez-Sacristan, A., Deu-Pons, J., Centeno, E., Garcia-Garcia, J., Sanz, F. and Furlong, L.I. (2017) DisGeNET: a comprehensive platform integrating information on human disease-associated genes and variants. *Nucleic Acids Res.*, **45**, D833-D839.
24. Gong, J., Mei, S., Liu, C., Xiang, Y., Ye, Y., Zhang, Z., Feng, J., Liu, R., Diao, L., Guo, A.Y. *et al.* (2018) PancanQTL: systematic identification of cis-eQTLs and trans-eQTLs in 33 cancer types. *Nucleic Acids Res.*, **46**, D971-D976.
25. Edgar, R., Domrachev, M. and Lash, A.E. (2002) Gene Expression Omnibus: NCBI gene expression and hybridization array data repository. *Nucleic Acids Res.*, **30**, 207-210.
26. Rocca-Serra, P., Brazma, A., Parkinson, H., Sarkans, U., Shojatalab, M., Contrino, S., Vilo, J., Abeygunawardena, N., Mukherjee, G., Holloway, E. *et al.* (2003) ArrayExpress: a public database of gene expression data at EBI. *C. R. Biol.*, **326**, 1075-1078.
27. Ponten, F., Schwenk, J.M., Asplund, A. and Edqvist, P.H. (2011) The Human Protein Atlas as a proteomic resource for biomarker discovery. *J. Intern. Med.*, **270**, 428-446.
28. Kim, M.S., Pinto, S.M., Getnet, D., Nirujogi, R.S., Manda, S.S., Chaerkady, R., Madugundu, A.K., Kelkar, D.S., Isserlin, R., Jain, S. *et al.* (2014) A draft map of the human proteome. *Nature*, **509**, 575-581.
29. Smith, C.M., Finger, J.H., Hayamizu, T.F., McCright, I.J., Xu, J., Berghout, J., Campbell, J., Corbani, L.E., Forthofer, K.L., Frost, P.J. *et al.* (2014) The mouse Gene Expression Database (GXD): 2014 update. *Nucleic Acids Res.*, **42**, D818-824.
30. Kim, P., Park, A., Han, G., Sun, H., Jia, P. and Zhao, Z. (2018) TissGDB: tissue-specific gene database in cancer. *Nucleic Acids Res.*, **46**, D1031-D1038.
31. Zhang, Z. and Townsend, J.P. (2010) The filamentous fungal gene expression database (FFGED). *Fungal Genet. Biol.*, **47**, 199-204.
32. Wu, Y., Yao, Y.G. and Luo, X.J. (2017) SZDB: A Database for Schizophrenia Genetic Research. *Schizophr. Bull.*, **43**, 459-471.
33. Palasca, O., Santos, A., Stolte, C., Gorodkin, J. and Jensen, L.J. (2018) TISSUES 2.0: an integrative web resource on mammalian tissue expression. *Database (Oxford)*, **2018**.
34. Grillo, G., Turi, A., Licciulli, F., Mignone, F., Liuni, S., Banfi, S., Gennarino, V.A., Horner, D.S., Pavesi, G., Picardi, E. *et al.* (2010) UTRdb and UTRsite (RELEASE 2010): a collection of sequences and regulatory motifs of the untranslated regions of eukaryotic mRNAs. *Nucleic Acids Res.*, **38**, D75-80.
35. Glazar, P., Papavasileiou, P. and Rajewsky, N. (2014) circBase: a database for circular RNAs. *RNA*, **20**, 1666-1670.
36. Chen, X., Han, P., Zhou, T., Guo, X., Song, X. and Li, Y. (2016) circRNADb: A comprehensive database for human circular RNAs with protein-coding annotations. *Sci. Rep.*, **6**, 34985.
37. Liu, Y.C., Li, J.R., Sun, C.H., Andrews, E., Chao, R.F., Lin, F.M., Weng, S.L., Hsu, S.D., Huang, C.C., Cheng, C. *et al.* (2016) CircNet: a database of circular RNAs derived from transcriptome sequencing data. *Nucleic Acids Res.*, **44**, D209-215.
38. Ghosal, S., Das, S., Sen, R., Basak, P. and Chakrabarti, J. (2013) Circ2Traits: a comprehensive database for circular RNA potentially associated with disease and traits. *Front. Genet.*, **4**, 283.
39. Chou, C.H., Chang, N.W., Shrestha, S., Hsu, S.D., Lin, Y.L., Lee, W.H., Yang, C.D., Hong, H.C., Wei, T.Y., Tu, S.J. *et al.* (2016) miRTarBase 2016: updates to the experimentally validated miRNA-target interactions database. *Nucleic Acids Res.*, **44**, D239-247.
40. Betel, D., Wilson, M., Gabow, A., Marks, D.S. and Sander, C. (2008) The microRNA.org resource: targets and expression. *Nucleic Acids Res.*, **36**, D149-153.
41. Matys, V., Kel-Margoulis, O.V., Fricke, E., Liebich, I., Land, S., Barre-Dirrie, A., Reuter, I., Chekmenev, D., Krull, M., Hornischer, K. *et al.* (2006) TRANSFAC and its module TRANSCompel: transcriptional gene regulation in eukaryotes. *Nucleic Acids Res.*, **34**, D108-110.
42. Dweep, H. and Gretz, N. (2015) miRWalk2.0: a comprehensive atlas of microRNA-target interactions. *Nat. methods*, **12**, 697.
43. Agarwal, V., Bell, G.W., Nam, J.W. and Bartel, D.P. (2015) Predicting effective microRNA target sites in mammalian mRNAs. *Elife*, **4**.
44. Xiao, F., Zuo, Z., Cai, G., Kang, S., Gao, X. and Li, T. (2009) miRecords: an integrated resource for microRNA-target interactions. *Nucleic Acids Res.*, **37**, D105-110.
45. Hsu, S.D., Chu, C.H., Tsou, A.P., Chen, S.J., Chen, H.C., Hsu, P.W., Wong, Y.H., Chen, Y.H., Chen, G.H. and Huang, H.D. (2008) miRNAMap 2.0: genomic maps of microRNAs in metazoan genomes. *Nucleic Acids Res.*, **36**, D165-169.
46. Bhattacharya, A. and Cui, Y. (2016) SomamiR 2.0: a database of cancer somatic mutations altering microRNA-ceRNA interactions. *Nucleic Acids Res.*, **44**, D1005-1010.
47. Jeggari, A., Marks, D.S. and Larsson, E. (2012) miRcode: a map of putative microRNA target sites in the long non-coding transcriptome. *Bioinformatics*, **28**, 2062-2063.
48. Yi, Y., Zhao, Y., Li, C., Zhang, L., Huang, H., Li, Y., Liu, L., Hou, P., Cui, T., Tan, P. *et al.* (2017) RAID v2.0: an updated resource of RNA-associated interactions across organisms. *Nucleic Acids Res.*, **45**, D115-D118.
49. Chen, G., Wang, Z., Wang, D., Qiu, C., Liu, M., Chen, X., Zhang, Q., Yan, G. and Cui, Q. (2013) LncRNADisease: a database for long-non-coding RNA-associated diseases. *Nucleic Acids Res.*, **41**, D983-986.
50. Rosikiewicz, W., Suzuki, Y. and Makalowska, I. (2018) OverGeneDB: a database of 5' end protein coding overlapping genes in human and mouse genomes. *Nucleic Acids Res.*, **46**, D186-D193.
51. Wei, Y., Zhang, S., Shang, S., Zhang, B., Li, S., Wang, X., Wang, F., Su, J., Wu, Q., Liu, H. *et al.* (2016) SEA: a super-enhancer archive. *Nucleic Acids Res.*, **44**, D172-179.
52. He, X., Chang, S., Zhang, J., Zhao, Q., Xiang, H., Kusonmano, K., Yang, L., Sun, Z.S., Yang, H. and Wang, J. (2008) MethyCancer: the database of human DNA methylation and cancer. *Nucleic Acids Res.*, **36**, D836-841.
53. Das, J. and Yu, H. (2012) HINT: High-quality protein interactomes and their applications in understanding human disease. *BMC Syst. Biol.*, **6**, 92.
54. Cowley, M.J., Pinese, M., Kassahn, K.S., Waddell, N., Pearson, J.V., Grimmond, S.M., Biankin, A.V., Hautaniemi, S. and Wu, J. (2012) PINA v2.0: mining interactome modules. *Nucleic Acids Res.*, **40**, D862-865.
55. Calderone, A., Castagnoli, L. and Cesareni, G. (2013) mentha: a resource for browsing integrated protein-interaction networks. *Nat. methods*, **10**, 690-691.
56. Li, T., Wernersson, R., Hansen, R.B., Horn, H., Mercer, J., Slodkowicz, G., Workman, C.T., Rigina, O., Rapacki, K., Staerfeldt, H.H. *et al.* (2017) A scored human protein-protein interaction network to catalyze genomic interpretation. *Nat. methods*, **14**, 61-64.
57. Hu, Y., Vinayagam, A., Nand, A., Comjean, A., Chung, V., Hao, T., Mohr, S.E. and Perrimon, N. (2018) Molecular Interaction Search Tool (MIST): an integrated resource for mining gene and protein interaction data. *Nucleic Acids Res.*, **46**, D567-D574.
58. Kotlyar, M., Pastrello, C., Sheahan, N. and Jurisica, I. (2016) Integrated interactions database: tissue-specific view of the human and model organism interactomes. *Nucleic Acids Res.*, **44**, D536-541.
59. Razick, S., Magklaras, G. and Donaldson, I.M. (2008) iRefIndex: a consolidated protein interaction database with provenance. *BMC Bioinformatics*, **9**, 405.
60. Gong, J., Shao, D., Xu, K., Lu, Z., Lu, Z.J., Yang, Y.T. and Zhang, Q.C. (2018) RISE: a database of RNA interactome from sequencing experiments. *Nucleic Acids Res.*, **46**, D194-D201.
61. Basha, O., Shpringer, R., Argov, C.M. and Yeger-Lotem, E. (2018) The DifferentialNet database of differential protein-protein interactions in human tissues. *Nucleic Acids Res.*, **46**, D522-D526.
62. Han, H., Cho, J.W., Lee, S., Yun, A., Kim, H., Bae, D., Yang, S., Kim, C.Y., Lee, M., Kim, E. *et al.* (2018) TRRUST v2: an expanded reference database of human and mouse transcriptional regulatory interactions. *Nucleic Acids Res.*, **46**, D380-D386.
63. Higueruelo, A.P., Jubb, H. and Blundell, T.L. (2013) TIMBAL v2: update of a database holding small molecules modulating protein-protein interactions. *Database (Oxford)*, **2013**, bat039.
64. Gilson, M.K., Liu, T., Baitaluk, M., Nicola, G., Hwang, L. and Chong, J. (2016) BindingDB in 2015: A public database for medicinal chemistry, computational chemistry and systems pharmacology. *Nucleic Acids Res.*, **44**, D1045-1053.
65. Anand, P., Nagarajan, D., Mukherjee, S. and Chandra, N. (2014) PLIC: protein-ligand interaction clusters. *Database (Oxford)*, **2014**, bau029.
66. Junge, A., Refsgaard, J.C., Garde, C., Pan, X., Santos, A., Alkan, F., Anthon, C., von Mering, C., Workman, C.T., Jensen, L.J. *et al.* (2017) RAIN: RNA-protein Association and Interaction Networks. *Database (Oxford)*, **2017**.
67. Yang, T.H., Wang, C.C., Wang, Y.C. and Wu, W.S. (2014) YTRP: a repository for yeast transcriptional regulatory pathways. *Database (Oxford)*, **2014**, bau014.
68. Liu, Z.P., Wu, C., Miao, H. and Wu, H. (2015) RegNetwork: an integrated database of transcriptional and post-transcriptional regulatory networks in human and mouse. *Database (Oxford)*, **2015**.
69. Zhu, F., Shi, Z., Qin, C., Tao, L., Liu, X., Xu, F., Zhang, L., Song, Y., Liu, X., Zhang, J. *et al.* (2012) Therapeutic target database update 2012: a resource for facilitating target-oriented drug discovery. *Nucleic Acids Res.*, **40**, D1128-1136.
70. Law, V., Knox, C., Djoumbou, Y., Jewison, T., Guo, A.C., Liu, Y., Maciejewski, A., Arndt, D., Wilson, M., Neveu, V. *et al.* (2014) DrugBank 4.0: shedding new light on drug metabolism. *Nucleic Acids Res.*, **42**, D1091-1097.
71. Zhang, J.X., Huang, W.J., Zeng, J.H., Huang, W.H., Wang, Y., Zhao, R., Han, B.C., Liu, Q.F., Chen, Y.Z. and Ji, Z.L. (2007) DITOP: drug-induced toxicity related protein database. *Bioinformatics*, **23**, 1710-1712.
72. Verbruggen, B., Gunnarsson, L., Kristiansson, E., Osterlund, T., Owen, S.F., Snape, J.R. and Tyler, C.R. (2018) ECOdrug: a database connecting drugs and conservation of their targets across species. *Nucleic Acids Res.*, **46**, D930-D936.
73. Cotto, K.C., Wagner, A.H., Feng, Y.Y., Kiwala, S., Coffman, A.C., Spies, G., Wollam, A., Spies, N.C., Griffith, O.L. and Griffith, M. (2018) DGIdb 3.0: a redesign and expansion of the drug-gene interaction database. *Nucleic Acids Res.*, **46**, D1068-D1073.
74. Pawson, A.J., Sharman, J.L., Benson, H.E., Faccenda, E., Alexander, S.P., Buneman, O.P., Davenport, A.P., McGrath, J.C., Peters, J.A., Southan, C. *et al.* (2014) The IUPHAR/BPS Guide to PHARMACOLOGY: an expert-driven knowledgebase of drug targets and their ligands. *Nucleic Acids Res.*, **42**, D1098-1106.
75. Gao, Z., Li, H., Zhang, H., Liu, X., Kang, L., Luo, X., Zhu, W., Chen, K., Wang, X. and Jiang, H. (2008) PDTD: a web-accessible protein database for drug target identification. *BMC Bioinformatics*, **9**, 104.
76. Davis, A.P., Wiegers, T.C., Roberts, P.M., King, B.L., Lay, J.M., Lennon-Hopkins, K., Sciaky, D., Johnson, R., Keating, H., Greene, N. *et al.* (2013) A CTD-Pfizer collaboration: manual curation of 88,000 scientific articles text mined for drug-disease and drug-phenotype interactions. *Database (Oxford)*, **2013**, bat080.
77. Berman, H.M., Westbrook, J., Feng, Z., Gilliland, G., Bhat, T.N., Weissig, H., Shindyalov, I.N. and Bourne, P.E. (2000) The Protein Data Bank. *Nucleic Acids Res.*, **28**, 235-242.
78. Madej, T., Addess, K.J., Fong, J.H., Geer, L.Y., Geer, R.C., Lanczycki, C.J., Liu, C., Lu, S., Marchler-Bauer, A., Panchenko, A.R. *et al.* (2012) MMDB: 3D structures and macromolecular interactions. *Nucleic Acids Res.*, **40**, D461-464.
79. Andreeva, A., Howorth, D., Chothia, C., Kulesha, E. and Murzin, A.G. (2014) SCOP2 prototype: a new approach to protein structure mining. *Nucleic Acids Res.*, **42**, D310-314.
80. Xu, H., Zhou, J., Lin, S., Deng, W., Zhang, Y. and Xue, Y. (2017) PLMD: An updated data resource of protein lysine modifications. *J. Genet. Genomics*, **44**, 243-250.
81. Ullah, S., Lin, S., Xu, Y., Deng, W., Ma, L., Zhang, Y., Liu, Z. and Xue, Y. (2016) dbPAF: an integrative database of protein phosphorylation in animals and fungi. *Sci Rep.*, **6**, 23534.
82. Cheng, H., Deng, W., Wang, Y., Ren, J., Liu, Z. and Xue, Y. (2014) dbPPT: a comprehensive database of protein phosphorylation in plants. *Database (Oxford)*, **2014**, bau121.
83. Ren, J., Jiang, C., Gao, X., Liu, Z., Yuan, Z., Jin, C., Wen, L., Zhang, Z., Xue, Y. and Yao, X. (2010) PhosSNP for systematic analysis of genetic polymorphisms that influence protein phosphorylation. *Mol. Cell. Proteomics.*, **9**, 623-634.
84. Hornbeck, P.V., Zhang, B., Murray, B., Kornhauser, J.M., Latham, V. and Skrzypek, E. (2015) PhosphoSitePlus, 2014: mutations, PTMs and recalibrations. *Nucleic Acids Res.*, **43**, D512-520.
85. Huang, K.Y., Su, M.G., Kao, H.J., Hsieh, Y.C., Jhong, J.H., Cheng, K.H., Huang, H.D. and Lee, T.Y. (2016) dbPTM 2016: 10-year anniversary of a resource for post-translational modification of proteins. *Nucleic Acids Res.*, **44**, D435-446.
86. Goel, R., Harsha, H.C., Pandey, A. and Prasad, T.S. (2012) Human Protein Reference Database and Human Proteinpedia as resources for phosphoproteome analysis. *Mol. Biosyst.*, **8**, 453-463.
87. Dinkel, H., Chica, C., Via, A., Gould, C.M., Jensen, L.J., Gibson, T.J. and Diella, F. (2011) Phospho.ELM: a database of phosphorylation sites--update 2011. *Nucleic Acids Res.*, **39**, D261-267.
88. The UniProt Consortium. (2017) UniProt: the universal protein knowledgebase. *Nucleic Acids Res.*, **45**, D158-D169.
89. Gnad, F., Gunawardena, J. and Mann, M. (2011) PHOSIDA 2011: the posttranslational modification database. *Nucleic Acids Res.*, **39**, D253-260.
90. Chatr-Aryamontri, A., Oughtred, R., Boucher, L., Rust, J., Chang, C., Kolas, N.K., O'Donnell, L., Oster, S., Theesfeld, C., Sellam, A. *et al.* (2017) The BioGRID interaction database: 2017 update. *Nucleic Acids Res.*, **45**, D369-D379.
91. Gupta, R., Birch, H., Rapacki, K., Brunak, S. and Hansen, J.E. (1999) O-GLYCBASE version 4.0: a revised database of O-glycosylated proteins. *Nucleic Acids Res.*, **27**, 370-372.
92. Kreegipuu, A., Blom, N. and Brunak, S. (1999) PhosphoBase, a database of phosphorylation sites: release 2.0. *Nucleic Acids Res.*, **27**, 237-239.
93. Chen, T., Zhou, T., He, B., Yu, H., Guo, X., Song, X. and Sha, J. (2014) mUbiSiDa: a comprehensive database for protein ubiquitination sites in mammals. *PLoS One*, **9**, e85744.
94. Bernhofer, M., Goldberg, T., Wolf, S., Ahmed, M., Zaugg, J., Boden, M. and Rost, B. (2018) NLSdb-major update for database of nuclear localization signals and nuclear export signals. *Nucleic Acids Res.*, **46**, D503-D508.
95. Binder, J.X., Pletscher-Frankild, S., Tsafou, K., Stolte, C., O'Donoghue, S.I., Schneider, R. and Jensen, L.J. (2014) COMPARTMENTS: unification and visualization of protein subcellular localization evidence. *Database (Oxford)*, **2014**, bau012.
96. Li, S., Shui, K., Zhang, Y., Lv, Y., Deng, W., Ullah, S., Zhang, L. and Xue, Y. (2017) CGDB: a database of circadian genes in eukaryotes. *Nucleic Acids Res.*, **45**, D397-D403.
97. Deng, W., Ma, L., Zhang, Y., Zhou, J., Wang, Y., Liu, Z. and Xue, Y. (2018) THANATOS: an integrative data resource of proteins and post-translational modifications in the regulation of autophagy. *Autophagy*, **14**, 296-310.
98. Shah, A., Chen, D., Boda, A.R., Foster, L.J., Davis, M.J. and Hill, M.M. (2015) RaftProt: mammalian lipid raft proteome database. *Nucleic Acids Res.*, **43**, D335-338.
99. Wang, Y., Liu, Z., Cheng, H., Gao, T., Pan, Z., Yang, Q., Guo, A. and Xue, Y. (2014) EKPD: a hierarchical database of eukaryotic protein kinases and protein phosphatases. *Nucleic Acids Res.*, **42**, D496-502.
100. Manning, G., Whyte, D.B., Martinez, R., Hunter, T. and Sudarsanam, S. (2002) The protein kinase complement of the human genome. *Science*, **298**, 1912-1934.
101. Lu, Z. and Hunter, T. (2018) Metabolic Kinases Moonlighting as Protein Kinases. *Trends Biochem. Sci.*, **43**, 301-310.
102. Rigden, D.J. (2008) The histidine phosphatase superfamily: structure and function. *Biochem. J.*, **409**, 333-348.
103. Shi, Y. (2009) Serine/threonine phosphatases: mechanism through structure. *Cell*, **139**, 468-484.
104. Takeda, K., Komuro, Y., Hayakawa, T., Oguchi, H., Ishida, Y., Murakami, S., Noguchi, T., Kinoshita, H., Sekine, Y., Iemura, S. *et al.* (2009) Mitochondrial phosphoglycerate mutase 5 uses alternate catalytic activity as a protein serine/threonine phosphatase to activate ASK1. *Proc. Natl. Acad. Sci. USA*, **106**, 12301-12305.
105. Alonso, A., Nunes-Xavier, C.E., Bayon, Y. and Pulido, R. (2016) The Extended Family of Protein Tyrosine Phosphatases. *Methods Mol. Biol.*, **1447**, 1-23.
106. Chen, M.J., Dixon, J.E. and Manning, G. (2017) Genomics and evolution of protein phosphatases. *Sci. Signal.*, **10**.
107. Kilanczyk, E., Filipek, S. and Filipek, A. (2011) ERK1/2 is dephosphorylated by a novel phosphatase--CacyBP/SIP. *Biochem. Biophys. Res. Commun.*, **404**, 179-183.
108. Kilanczyk, E., Wasik, U. and Filipek, A. (2012) CacyBP/SIP phosphatase activity in neuroblastoma NB2a and colon cancer HCT116 cells. *Biochem. Cell Biol*, **90**, 558-564.
109. Zhuo, D.X., Zhang, X.W., Jin, B., Zhang, Z., Xie, B.S., Wu, C.L., Gong, K. and Mao, Z.B. (2013) CSTP1, a novel protein phosphatase, blocks cell cycle, promotes cell apoptosis, and suppresses tumor growth of bladder cancer by directly dephosphorylating Akt at Ser473 site. *PLoS One*, **8**, e65679.
110. Selengut, J.D. (2001) MDP-1 is a new and distinct member of the haloacid dehalogenase family of aspartate-dependent phosphohydrolases. *Biochemistry*, **40**, 12704-12711.
111. Moorhead, G.B., Trinkle-Mulcahy, L. and Ulke-Lemee, A. (2007) Emerging roles of nuclear protein phosphatases. *Nat. Rev. Mol. Cell Biol.*, **8**, 234-244.
112. Seifried, A., Knobloch, G., Duraphe, P.S., Segerer, G., Manhard, J., Schindelin, H., Schultz, J. and Gohla, A. (2014) Evolutionary and structural analyses of mammalian haloacid dehalogenase-type phosphatases AUM and chronophin provide insight into the basis of their different substrate specificities. *J. Biol. Chem.*, **289**, 3416-3431.
113. Vassylyev, D.G. and Symersky, J. (2007) Crystal structure of pyruvate dehydrogenase phosphatase 1 and its functional implications. *J. Mol. Biol.*, **370**, 417-426.
114. Gong, W., Zhou, D., Ren, Y., Wang, Y., Zuo, Z., Shen, Y., Xiao, F., Zhu, Q., Hong, A., Zhou, X. *et al.* (2008) PepCyber:P~PEP: a database of human protein protein interactions mediated by phosphoprotein-binding domains. *Nucleic Acids Res.*, **36**, D679-683.
115. Thakur, J.K., Yadav, A. and Yadav, G. (2014) Molecular recognition by the KIX domain and its role in gene regulation. *Nucleic Acids Res.*, **42**, 2112-2125.
116. Takahasi, K., Suzuki, N.N., Horiuchi, M., Mori, M., Suhara, W., Okabe, Y., Fukuhara, Y., Terasawa, H., Akira, S., Fujita, T. *et al.* (2003) X-ray crystal structure of IRF-3 and its functional implications. *Nat. Struct. Biol.*, **10**, 922-927.
117. Carty, S.M., Goldstrohm, A.C., Sune, C., Garcia-Blanco, M.A. and Greenleaf, A.L. (2000) Protein-interaction modules that organize nuclear function: FF domains of CA150 bind the phosphoCTD of RNA polymerase II. *Proc. Natl. Acad. Sci. USA*, **97**, 9015-9020.
118. Ernst, A., Appleton, B.A., Ivarsson, Y., Zhang, Y., Gfeller, D., Wiesmann, C. and Sidhu, S.S. (2014) A structural portrait of the PDZ domain family. *J. Mol. Biol.*, **426**, 3509-3519.
119. Morishige, M., Hashimoto, S., Ogawa, E., Toda, Y., Kotani, H., Hirose, M., Wei, S., Hashimoto, A., Yamada, A., Yano, H. *et al.* (2008) GEP100 links epidermal growth factor receptor signalling to Arf6 activation to induce breast cancer invasion. *Nat. Cell Biol.*, **10**, 85-92.
120. Mukherjee, M., Chow, S.Y., Yusoff, P., Seetharaman, J., Ng, C., Sinniah, S., Koh, X.W., Asgar, N.F., Li, D., Yim, D. *et al.* (2012) Structure of a novel phosphotyrosine-binding domain in Hakai that targets E-cadherin. *EMBO J.*, **31**, 1308-1319.
121. Yaffe, M.B. (2002) Phosphotyrosine-binding domains in signal transduction. *Nat. Rev. Mol. Cell Biol.*, **3**, 177-186.
122. Forman-Kay, J.D. and Pawson, T. (1999) Diversity in protein recognition by PTB domains. *Curr. Opin. Struct. Biol.*, **9**, 690-695.
123. Yaffe, M.B. and Elia, A.E. (2001) Phosphoserine/threonine-binding domains. *Curr. Opin.* *Cell Biol.*, **13**, 131-138.

**Supplementary Figure S1.** The performance evaluation of HMM identifications. For two PK families (PKA and CAMK1), one PP family (PP1) and one PPBD family (14-3-3), the ROC curves were drawn and AUC values were calculated for the self-consistency (Blue lines) and LOO (Orange lines) validations. For each family, the *Ac*, *Sn* and *Sp* values under different log-odds scores were plotted, separately. The threshold value of each family was marked in a triangle.


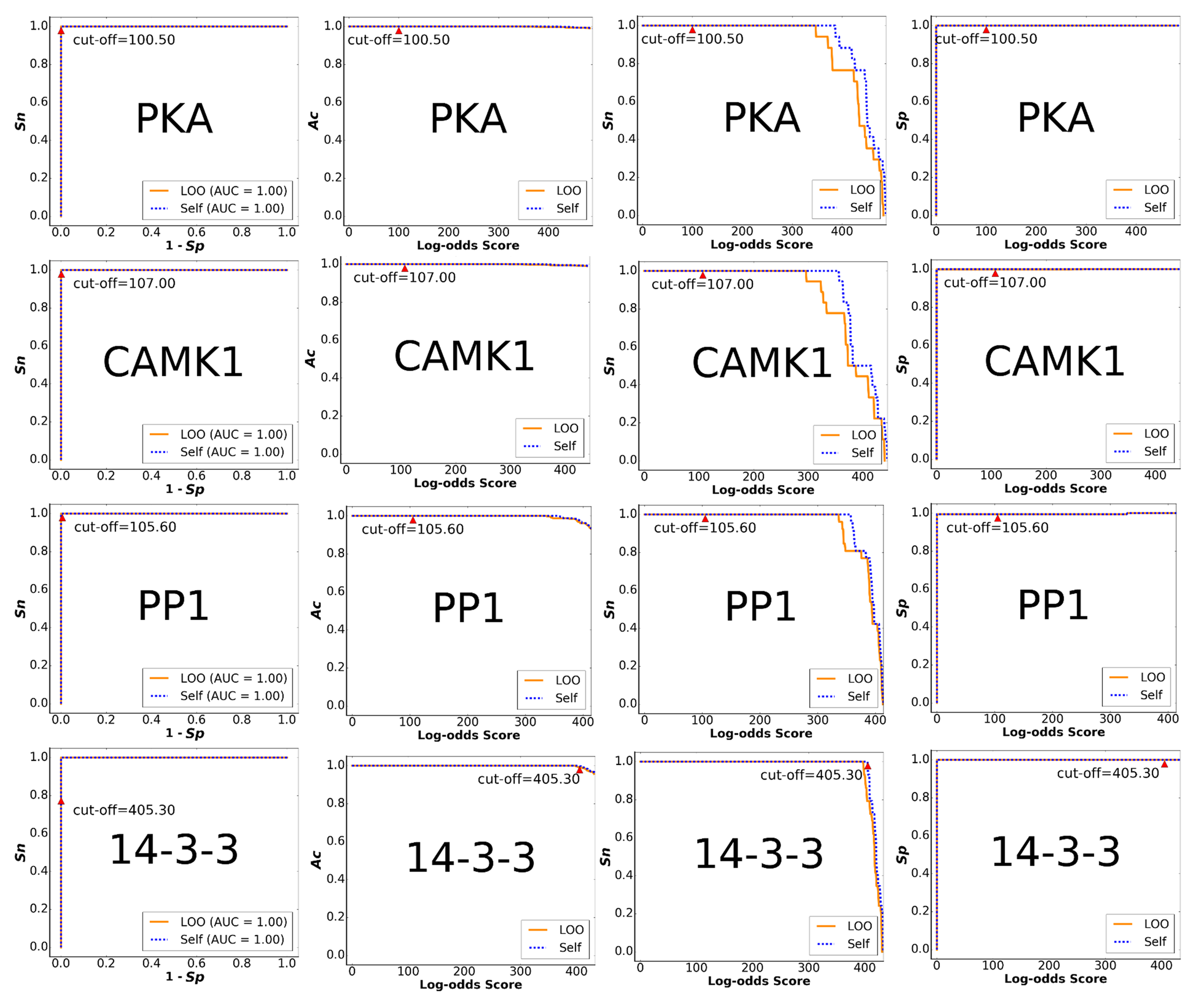


**Supplementary Figure S2.** The heatmap of classifications and numbers of 151 PK groups, 36 PP families and 21 PPBD families across 164 eukaryotes.


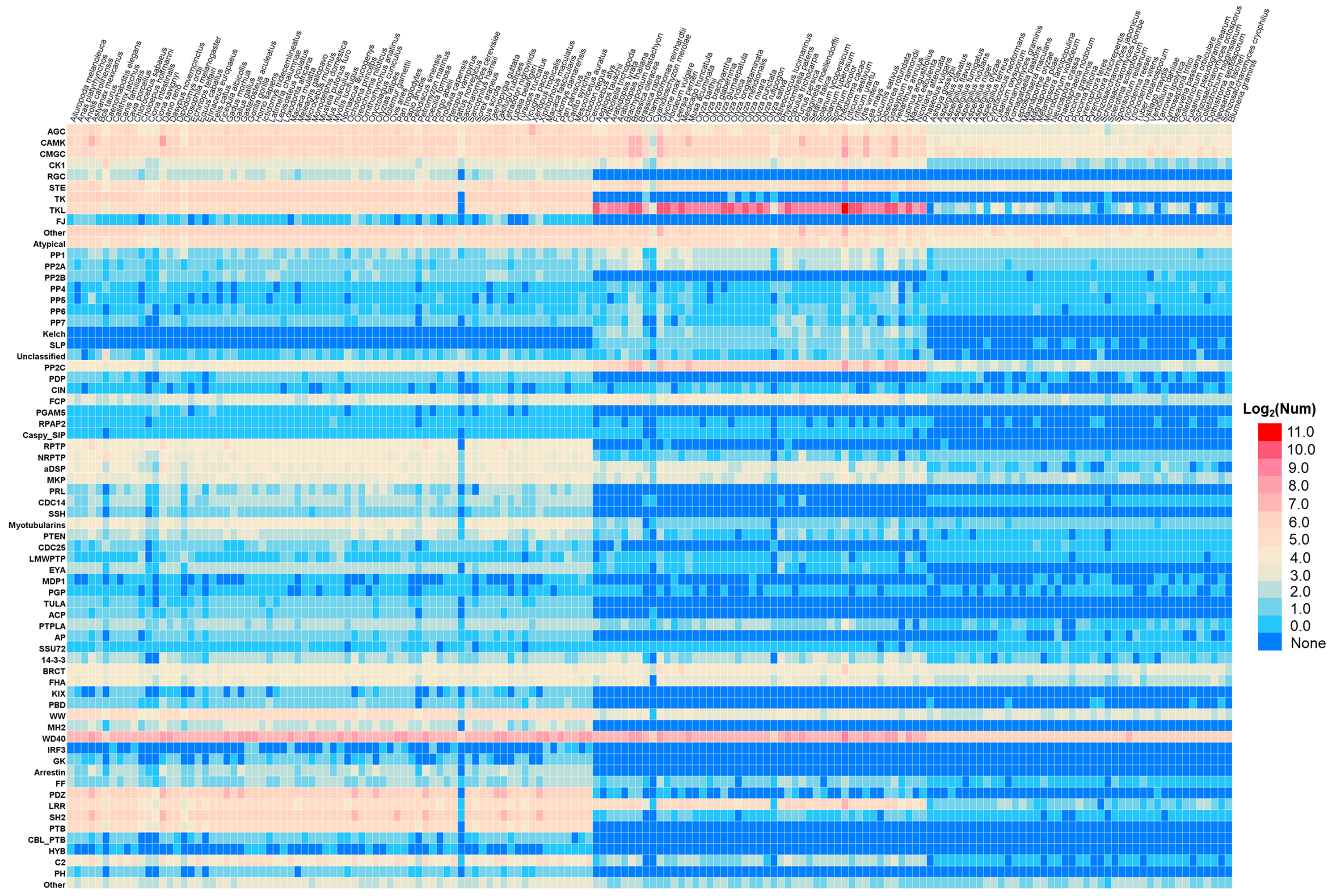


**Supplementary Figure S3.** (A) Distribution of cases with mutated MTOR across 20 cancers in TCGA. (B) Phosphorylation of residues S2448 and S2481 that are closely related multiple cancers. Data retrieved from PTMD (<http://ptmd.biocuckoo.org/>). (C) Average expression of MTOR in 37 cancers in TCGA. (D) Average DNA methylation level of MTOR in 36 cancers in TCGA.


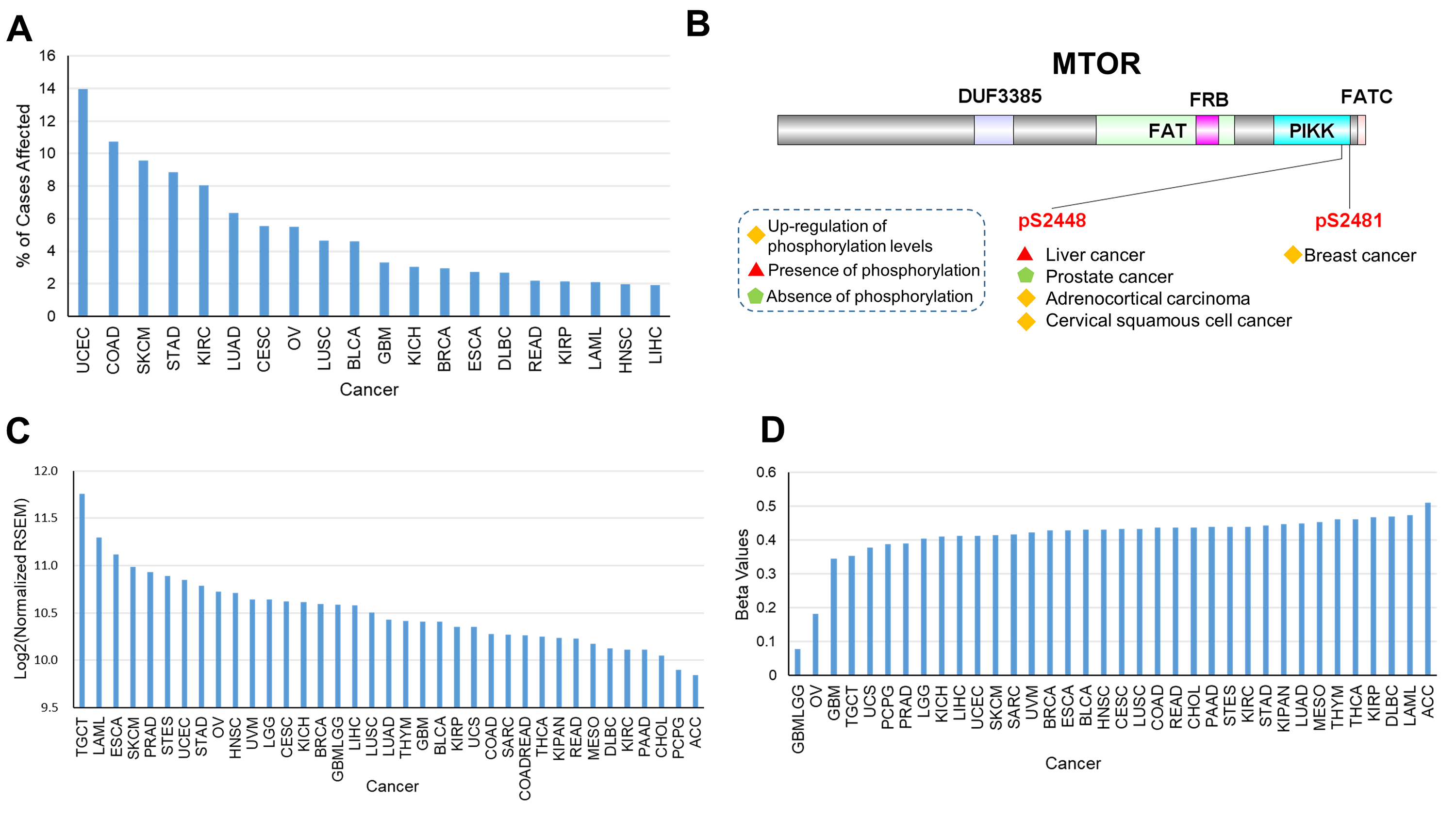


**Supplementary** **Tables**.

**Table S1.** A summary of public resources for the identification and classifications of kinases, phosphatases and PPBD-containing proteins. Kinannote is not a database but a computational tool for the prediction of eukaryotic PKs (ePKs) (19). *a*. *N/A*, not available.

**Table S2.** The data statistics of known PKs, PPs and PPBDs manually collected from the scientific literature. The basic information and corresponding references are also provided. In total, we acquired 2643 experimentally verified phospho-regulators, including 1,860 PKs, 439 PPs and 400 PPBD-containing proteins.

**Table S3.** The results of the self-consistency and LOO validations under the manually selected log-odds threshold values for 176 families. *Ac*, *Sn*, *Sp* and *MCC* values were calculated and presented.

**Table S4.** The detailed classifications and data statistics of PKs in iEKPD 2.0. In summary, 109,912 PKs in 164 eukaryotes were classified into 151 families.

**Table S5.** The detailed classifications and data statistics of PPs in iEKPD 2.0. In total, 23,294 PPs in 164 eukaryotes were classified into 36 families.

**Table S6.** The detailed classifications and data statistics of PPBDs in iEKPD 2.0. In total, 68,748 PPBD-containing proteins in 164 eukaryotes were classified into 21 families.

**Table S7.** The 100 public databases covering 13 aspects of the data. We mapped all regulators in 8 model species to these databases to provide a more comprehensive annotation in iEKPD 2.0. Moreover, PTM sites in UniProt were integrated with the exception of sites annotated with “By similarity”, “Potential” or “Probable”.

**Table S8.** Comparison between EKPD 1.0 and iEKPD 2.0.
